# Bacterial Recruitment to Carnivorous Pitcher Plant Communities: Identifying Sources Influencing Plant Microbiome Composition and Function

**DOI:** 10.3389/fmicb.2022.791079

**Published:** 2022-03-14

**Authors:** Jacob J. Grothjan, Erica B. Young

**Affiliations:** ^1^Department of Biological Sciences, University of Wisconsin-Milwaukee, Milwaukee, WI, United States; ^2^School of Freshwater Sciences, University of Wisconsin-Milwaukee, Milwaukee, WI, United States

**Keywords:** microbiome function, *Sarracenia purpurea*, carnivorous plant, bioinformatics, nutrient transformation, hydrolytic enzymes

## Abstract

Processes influencing recruitment of diverse bacteria to plant microbiomes remain poorly understood. In the carnivorous pitcher plant *Sarracenia purpurea* model system, individual pitchers open to collect rainwater, invertebrates and a diverse microbial community, and this detrital food web is sustained by captured insect prey. This study examined how potential sources of bacteria affect the development of the bacterial community within pitchers, how the host plant tissue affects community development and how established vs. assembling communities differ. In a controlled greenhouse experiment, seven replicate pitchers were allocated to five treatments to exclude specific bacterial sources or host tissue: milliQ water only, milliQ + insect prey, rainwater + prey, established communities + prey, artificial pitchers with milliQ + prey. Community composition and functions were examined over 8–40 weeks using bacterial gene sequencing and functional predictions, measurements of cell abundance, hydrolytic enzyme activity and nutrient transformations. Distinct community composition and functional differences between artificial and real pitchers confirm an important influence of host plant tissue on community development, but also suggest this could be partially related to host nutrient uptake. Significant recruitment of bacteria to pitchers from air was evident from many taxa common to all treatments, overlap in composition between milliQ, milliQ + prey, and rainwater + prey treatments, and few taxa unique to milliQ only pitchers. Community functions measured as hydrolytic enzyme (chitinase, protease) activity suggested a strong influence of insect prey additions and were linked to rapid transformation of insect nutrients into dissolved and inorganic sources. Bacterial taxa found in 6 of 7 replicate pitchers within treatments, the “core microbiome” showed tighter successional trajectories over 8 weeks than all taxa. Established pitcher community composition was more stable over 8 weeks, suggesting a diversity-stability relationship and effect of microinvertebrates on bacteria. This study broadly demonstrates that bacterial composition in host pitcher plants is related to both stochastic and specific bacterial recruitment and host plants influence microbial selection and support microbiomes through capture of insect prey.

## Introduction

The processes defining how host-associated microbial communities develop and assemble are complex and not well understood. Carnivorous pitcher plants host complex food webs and microbial communities, so have served as model systems for microbial and food web ecology, and the microbial roles in biogeochemical nutrient cycling (Ellison and Adamec, 2018; Young et al., 2018; Bittleston et al., 2021). The purple pitcher plant, *Sarracenia purpurea* subsp. *purpurea* L. uses carnivory to supplement mineral nutrition by capturing insect prey in modified leaves formed into pitchers. Initially the hollow pitchers are sealed and sterile, opening to fill with rainwater, and then recruit invertebrates and microbes from the environment (Newell and Nastase, 1998; Peterson et al., 2008). Insect prey is lured into the pitcher trap where they drown and are degraded by the invertebrates and bacterial activity, but the recruitment of bacteria into these novel communities is not well understood.

The eukaryotic and bacterial food web members play different roles in shredding and digesting the prey, releasing nutrients for host plant uptake. The presence of eukaryotic taxa in the food web can vary between habitats (Gray, 2012; Grothjan and Young, 2019) and can depend on the presence of different predator taxa (Gebühr et al., 2006; Paisie et al., 2014). The larger invertebrate organisms in these food webs have been well documented (Trzcinski et al., 2005; Gotelli and Ellison, 2006; Hoekman, 2007; Baiser et al., 2013) and more recently comprehensive eukaryotic diversity has been examined with DNA sequencing (TerHorst, 2011; Satler et al., 2016; Grothjan and Young, 2019). More broadly in food web ecology, the contribution of microbial diversity has rarely been fully incorporated into food web structure and function, though mass sequencing to define microbial diversity is helping to address this gap (Pringle and Hutchinson, 2020). In pitcher plants, sequencing has also provided a detailed appreciation for microbial diversity and functions in the food web (Boynton, 2012; Gray et al., 2012; Paisie et al., 2014; Armitage, 2017; Bittleston et al., 2018; Canter et al., 2018; Grothjan and Young, 2019). For this model system to help us understand microbial functions in food web ecology, and for broader understanding of plant host microbiome associations, we need more information about how microbial taxa are recruited to pitcher plant communities, and which sources of bacteria are most important in contributing functionally important taxa (Siragusa et al., 2007; Koopman and Carstens, 2011; Gray et al., 2012).

The microbial composition in pitcher plant fluid differs from the surrounding soil (Koopman et al., 2010; Bittleston et al., 2018) and therefore bacteria are likely recruited from a range of sources after pitcher opening. From early studies, it was considered that bacteria within pitchers are introduced with captured prey (Bradshaw and Creelman, 1984) or with colonization by invertebrates (Heard, 1994). With more recent genetic tools, hypotheses about bacterial recruitment can be more rigorously tested and alternative potential sources of bacteria to pitcher plant fluid can be examined. These sources include the captured prey (both external surfaces and gut contents), and the colonizing invertebrates, but also the surrounding air, rainwater, and plant debris inputs.

Pitchers are micro-habitats (typically < 50 mL in volume) that can host over 165 eukaryotic species (Adlassnig et al., 2011) but microbial taxa can be orders of magnitude higher (Grothjan and Young, 2019). Pitcher plants and other container plants like tank bromeliads have often been considered inert “vessels” for the community (Frank and Lounibos, 2009). However, studies with “artificial pitchers” suggest that food web composition and prey acquisition can be influenced by the host plant (Bittleston et al., 2018; Ellison et al., 2021). Food web member behaviors may also be important, as *Diptera* avoided artificial pitcher tubes for egg-laying (Ellison et al., 2021). Microbial phosphatase production involved in prey digestion was higher within living pitchers than in artificial tubes (Luciano and Newell, 2017) indicating some food web functions are also influenced by the host plant tissue.

Investigation of the *Sarracenia* invertebrate food web structure and function suggests convergence of diversity and taxonomic composition over the growing season (Miller and TerHorst, 2012). In the *Sarracenia* microbial communities, functions can vary across geographically separated populations of pitchers (Bittleston et al., 2016, 2021; Grothjan and Young, 2019; Freedman et al., 2021) and over time in *Darlingtonia* pitcher plants (Armitage, 2017). However, the roles of different bacterial sources, including rainwater, air, and prey capture, contributing functionally important taxa to the assemblages remains understudied in the *Sarracenia* microbial food web.

To address the gaps in our understanding of microbial recruitment to pitcher communities, this study examined communities hosted by *S. purpurea* pitchers, in a controlled greenhouse environment, applying bacterial gene sequencing and functional predictions, measures of bacterial cell abundance and hydrolytic enzyme activity, to address three key research questions:

1. How do potential sources of bacteria (air, rain, captured prey) affect the development of the bacterial community within pitchers? This was examined as changes in community composition in pitcher treatments which excluded different potential sources of bacteria, comparing with an established microbial community from field plants.
2. Does the host plant pitcher tissue affect the development of the bacterial community? This was examined by comparing communities in real and “artificial” pitchers placed close to the plants.
3. During development of microbial communities within pitchers, are different bacterial taxa contributed by different sources? This was examined by comparing community composition and functions including hydrolytic enzymes and nutrient transformations, in treatments excluding different potential bacterial sources.

## Materials and Methods

*Sarracenia purpurea* subsp. *purpurea* pitcher plants were collected from Cedarburg Bog (43°23.2′N, 88°0.63′W) a peatland fen described previously (Bott et al., 2008; Grothjan and Young, 2019). Plants in the field were selected and transferred to greenhouse cultivation. This procedure and greenhouse conditions are described in Supplementary Material.

### Pitcher Selection and Experimental Treatments

In the greenhouse, across 15 plants, 28 healthy young pitchers with at least 30 mL capacity were selected. Pitchers were emptied, sterilized briefly with 10% hypochlorite solution (Bott et al., 2008) rinsed four times with milliQ water and labeled. To examine the effect of the potential bacterial sources of rainwater, insect prey, and surrounding air on bacterial recruitment and pitcher community composition, pitchers were randomly assigned to one of four treatments: (1) milliQ water only (MQ), (2) milliQ water + prey addition (MQP), (3) rainwater + prey addition (RWP), and (4) pitcher fluid from field pitchers with established communities + prey addition (EST). Pitcher water added to pitchers for the established community was filtered (153 μm mesh, Sefar NITEX, Montreal) to remove larger food web invertebrates and detritus, but smaller particles and microbes were distributed among the established treatment pitchers. Rainwater was collected at Cedarburg Bog during May–June, 2015, filtered (153 μm mesh) and stored at 4°C until pitcher addition. (5) An additional treatment tested the effect of the host plant with “artificial pitchers” (ART) created with 50 mL polypropylene falcon tubes wrapped in black tape to reduce light and inserted into the sphagnum alongside experimental pitchers and filled with milliQ water + prey addition. There were seven replicate pitchers for each of the five treatments.

Invertebrate prey was added to the pitchers at regular intervals and collected at Cedarburg Bog via a suction trap and net, and taxa identified as ants (*Formicidae*), spiders (*Araneae*), flies (*Diptera*), and leafhoppers (*Homoptera*) and stored at −20°C. Prey were thawed, weighed, and coarsely chopped with a sterile razor blade before addition to pitchers. Each prey addition pitcher received two prey organisms on day 0 (1 ant, 1 spider), day 7 (1 ant, 1 fly), day 28 (1 fly, 1 leafhopper), and day 55 (1 fly, 1 spider).

### Sampling and Sample Processing

Bacterial community composition was monitored in pitcher water samples over the first 8 weeks during which most changes are expected (Armitage, 2017) and prey was added regularly, but the community functions were monitored for the full 44 weeks. Pitcher water samples were collected at regular time intervals from pitchers over the course of 314 days, starting August 2015 (Supplementary Table 1). Fluid was mixed and then collected with a sterile syringe and silicone tubing. Large detritus was returned to the pitcher and samples transferred to sterile collection tubes (Young et al., 2018). Sterile milliQ water was added to pitchers to replace the sampled volume. Samples were stored on ice and transported to the lab for prefiltering (153 μm mesh), and aliquoting for bacterial counts via epifluorescence, hydrolytic enzyme activity assays, community DNA extraction, and dissolved nutrient concentrations (Supplementary Table 1).

Pitcher fluid samples were assayed for activity of phosphatase, chitinase, and protease with microplate well assays using fluorescent substrates as described previously (Young et al., 2018), except that protease assays used L-leucine 2-naphthylamide substrate (Sarath et al., 1989). Samples for nutrient concentration analysis were filtered (Whatman GF/F) and the filtrate frozen at −20°C. Dissolved nitrogen (N) and phosphorus (P) concentrations were assayed on filtrates from samples collected on days 14 and 42, using methods adapted and tested for small volumes (1 mL per nutrient). Ammonium was assayed using the phenol-hypochlorite method (Parsons et al., 1984). Nitrate and total dissolved nitrogen (TDN) were assayed as nitrite following reduction using small-batch spongy cadmium method (Jones, 1984) with TDN reduction following potassium persulfate oxidation (Parsons et al., 1984). Soluble molybdate-reactive phosphorus (SRP) was assayed using the ammonium molybdate method and total dissolved phosphorus (TDP) assayed as SRP following high temperature potassium persulfate digestion (Young et al., 2018). All reacted samples were read in 96-well plate with an absorbance reader.

### DNA and Sequencing

For community DNA analysis, particles were vacuum-filtered onto 0.2 μm polycarbonate membrane filters (GVS Life Sciences) and filters stored at −70°C. DNA was extracted from filters using FAST DNA soil extraction kit (MP Biomedicals). DNA concentration and purity were confirmed with agarose gel electrophoresis and spectrophotometry (NanoDrop ND-1000). Bacterial 16S rRNA sequences were targeted with PCR using 16S rRNA V3_4 primers 341F: CCTACGGGNGGCWGCAG and 805R: GACTACHVGGGTATCTAATCC (Klindworth et al., 2013). Samples were sequenced by UW Madison Biotechnology Center using 2 × 300 bp sequencing runs on Illumina MiSeq equipment with 16S primers including Illumina adapters.

### Sequence Analysis

The 16S rRNA V3_4 region of sequences was analyzed using mothur software version 1.42.2 with the MiSeq SOP (Kozich et al., 2013). Sequences were aligned and matched against SILVA database Release 132 (Quast et al., 2013). To generate community composition, sequences were clustered into OTUs using the cluster.split command with 97% similarity OTU definition using the OptiClust algorithm in mothur (Westcott and Schloss, 2017). OTUs were then collapsed into family level taxonomy. Further bioinformatics processing details can be found within Grothjan and Young (2019). Of the total 8,939,254 sequences in samples, 1,966,332 sequences were removed from further analysis including singletons (only one sequence per OTU), mitochondrial taxa, chloroplast taxa, and Archaeal taxa due to low abundance, to more clearly visualize the bacterial families identified in different treatments.

Community analyses were carried out as described previously (Grothjan and Young, 2019) using mothur 1.42.2, QIIME (MacQIIME V1.9.1), PICRUSt 1.1.4. Community diversity values were calculated with mothur and QIIME. For sample relatedness trees, bootstrap values were generated in QIIME based on 100 iterations with a minimum of 75% of the smallest sample sequence number, and visualized in FigTree v1.4.4 (Rambaut, 2018). Community composition was also visualized using multivariate approaches in PAST V. 2.17C (Hammer et al., 2001). Community composition was also analyzed as the total sequences, which ranged from 247K to 511K per treatment per timepoint (replicates pooled). Sequences numbers were normalized to the median value across all treatments and timepoints.

The core microbiome can be defined as the most common taxa found across replicates within a treatment. The published criteria for “core microbiome” vary with both study system and publication, with “core” taxa representing 30–95% of the sequences (Huse et al., 2012; Ainsworth et al., 2015) though justifications for these differences are rare (Risely, 2020). We applied a numerical definition of the core microbiome as OTUs that were found in 85% of replicate samples within a treatment (Björk et al., 2017), or 6 out of 7 replicate pitchers per treatment. For each treatment, the taxa included in the core microbiome group was assembled from those identified in 6 out of 7 replicate pitchers at one or more timepoints. In some cases, this excluded OTUs which were very abundant in some replicates, but not present in ≥ 6 replicate samples.

Predictions of community functional capacities based on bacterial taxonomic composition were derived from 16S rRNA taxonomic information via the PICRUSt pipeline v1.1.4 (Langille et al., 2013). Community composition across replicate pitchers were pooled by treatment and data for NMDS, PCAs and cluster analysis which were generated through mothur and QIIME software, and visualized with PAST V. 2.17C. Metabolic prediction vectors used in the NMDS were reduced and condensed from the PICRUSt precalculated files which were based on normalized 16S copy number and KEGG ORTHOLOGY.

### Statistical Analysis

Statistical differences in abundance of particular taxa or bacterial composition between treatments were examined using ANOSIM in MacQIIME V1.9.1 (Allali et al., 2017). Treatment differences in enzyme activity, dissolved nutrient concentrations and bacterial taxon composition and diversity were also analyzed using linear mixed-effects models (lme) conducted in R using the nlme package (Pinheiro et al., 2021). Comparisons between sampling timepoints were considered as within subject (replicate pitcher) factors and comparisons between treatments as between subject factors. Planned orthogonal contrasts were constructed to test a reduced number of treatments or pooled treatments, to examine effects of the different bacterial sources. We included the following four orthogonal tests: (1) “MQP vs. RWP” treatments to test for the effect of water source. (2) “MQ vs. MQP” to test the effect of prey addition, (3) “ART vs. PIT” where PIT is the three pooled living pitcher treatments (MQ, MQP, RWP), to test the effect of pitcher tissue against artificial (ART) treatment. (4) “EST vs. AGE” to test the effect of a mature established community from the field (EST), against the three living pitcher assembling community treatments (MQ, MQP, RWP).

## Results

### Community Composition

**FIGURE 1:**
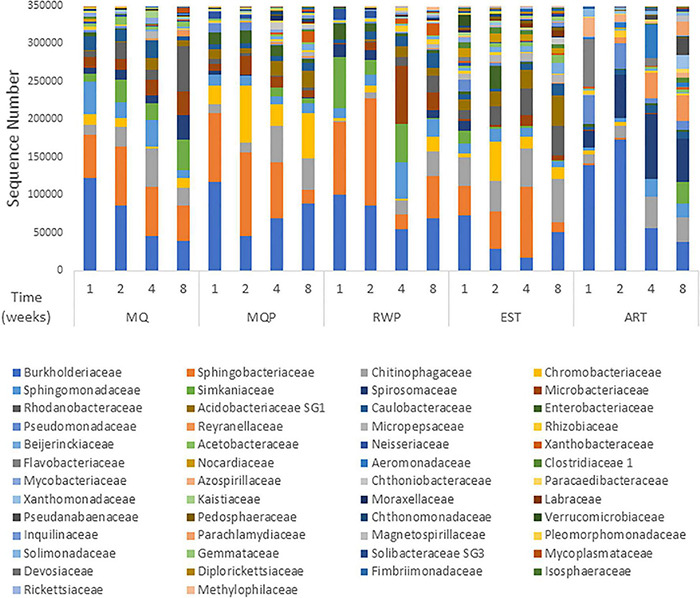
Bacterial family composition of pitcher communities within each treatment at each of the four sampling times based on 16S rRNA amplicon sequencing of total community DNA in a greenhouse experiment. Sequences per treatment were normalized to the median sequence value for all samples, then plotted by abundance rank. Treatments are milliQ only (MQ), milliQ + prey (MQP), rainwater + prey (RWP), established community (EST), and artificial pitchers (ART) with sampling timepoints at weeks 1, 2, 4, and 8. Each bar represents the total abundances in all seven replicate pitchers within each treatment. Sequence abundances of families representing < 0.1% were excluded.

**FIGURE 2:**
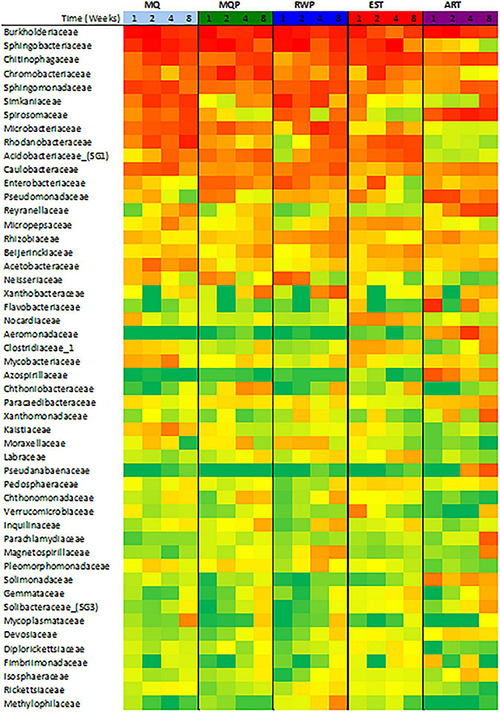
Heatmap of the 50 most abundant bacterial families identified in samples across four timepoints in the five treatments (see Figure 1). Values are the logarithmic transformation of each family abundance with the color scale showing green as zero abundance and red as the highest abundance.

**FIGURE 3:**
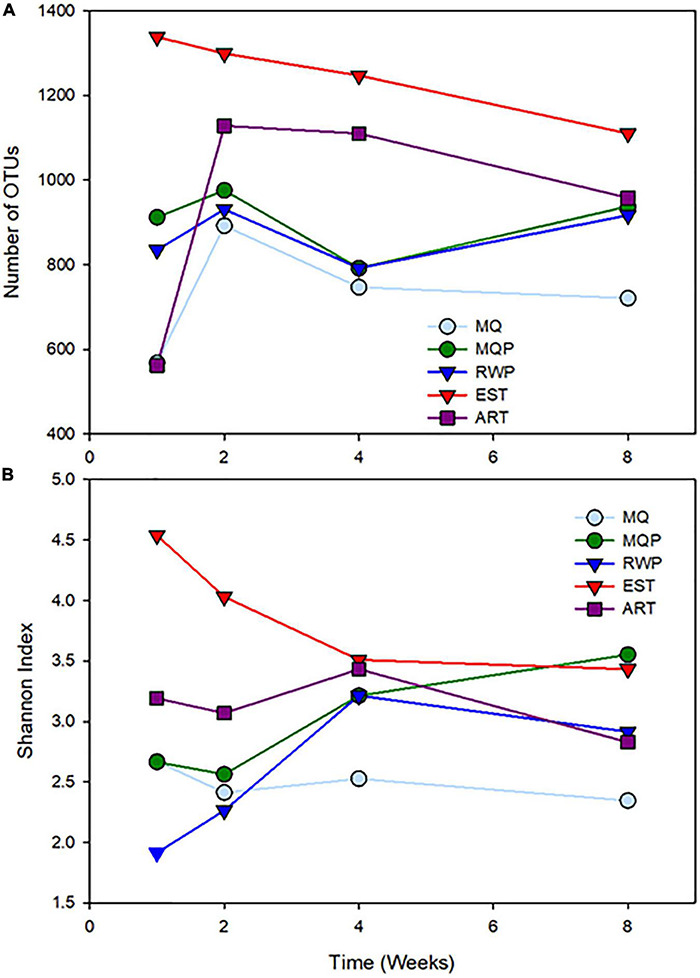
Changes in community diversity metrics, number of OTUs **(A)** and Shannon diversity index **(B)** in the five treatments over four timepoints.

Community composition varied with treatment and over time (Figure 1). Dominant bacterial families present in most treatments were *Burkholderiaceae*, *Sphingobacteriacaeae*, *Chitinophageaceae*, and *Chromobacteriaceae*, which accounted for 35–75% of all sequences in living pitchers (Figure 1). *Chitinophagaceae* was abundant in all treatments and many OTUs within the core microbiomes for each treatment were *Chitinophagaceae* including 62 OTUs in established (EST) pitchers and 14 OTUs in the MilliQ only (MQ) treatment. Artificial (ART) pitchers showed some distinct composition compared to living pitchers (Figure 2). *Aeromonadaceae*, *Pseudanabaenaceae*, *Reyranellaceae* were abundant in ART pitchers especially in weeks 4 and 8 but were absent or minimally present in other treatments; *Solimondaceae* were also more abundant in ART pitchers than in living pitcher treatments (ANOSIM *p* < 0.003). In contrast, *Sphingobacteriaceae* was a dominant taxon (up to 30% of sequences) in all living pitchers but was < 1% in ART pitchers. *Acidobacteriaceae* was common at most timepoints in living pitcher treatments but nearly absent from ART pitchers and *Microbacteriaceae* and *Rhodanobacteraceae* were more common in living pitcher treatments (*p* < 0.045, *p* < 0.001). Chloroplast sequences were removed from the composition shown in Figure 1 to better visualize the bacterial taxa, but sequences identified as chloroplast accounted for ∼50% of sequences in week 4 ART pitchers, but only ∼8% of sequences in living pitchers across other treatments and timepoints (data not shown). *Spiromonaceae* was also more common in ART pitchers (6–12% of sequences) but rarer (0.08–4.7%) in living pitchers especially in EST and MQP treatments (*p* < 0.002). ART pitchers also had significantly higher *Reyranellaceae* and *Solimonadaceae* OTUs (*p* < 0.009, 0.010). The ART treatment also showed some distinct taxon composition and diversity patterns over time to the assembling communities in living pitchers (MQ, MQP, RWP) (Figure 3).

Within the four living pitcher treatments, bacterial composition was more similar with the most differences between EST and other treatments. Over 50% of bacterial OTUs were common between replicate pitchers within the EST treatment, followed by the 40% of shared OTUs across pitchers in MQP (Supplementary Figure 1). An abundant *Microbacteriaceae* OTU was less common in EST than the other treatments (*p* < 0.006) while a *Nocardiaceae* OTU was more common in EST treatments (*p* < 0.001). *Spirosomaceae* became less abundant in MQP and EST treatments over time but not in MQ or RWP (Figure 2).

### Bacterial Diversity

The mature, established community from field pitchers had more OTUs than the other treatments (lme ANOVA *p* < 0.0001) (Figure 3). Full lme ANOVA statistical test results are in Supplementary Table 3. Rarefaction curves (Supplementary Figure 2), also showed EST pitchers with the highest number of sequences at all time points (Figure 3A). In week 2 and 4, ART had more OTUs than the living pitcher assembling communities (*p* < 0.035). In week 4, the MQP had more OTUs than the MQ treatment (*p* < 0.017). The EST pitchers also showed higher Shannon diversity (Figure 3B) than the other treatments (*p* < 0.001) and distinct differences at week 1 and 2 (*p* < 0.0015). At 2 weeks, the ART pitchers showed higher Shannon diversity than the living assembling communities (*p* < 0.045) and MQP showed higher Shannon diversity to MQ treatment (*p* < 0.0018). Good’s coverage estimates were high in all treatments (>0.98) indicating good sequencing depth (Supplementary Table 2).

### Sample Relatedness Trees

**FIGURE 4:**
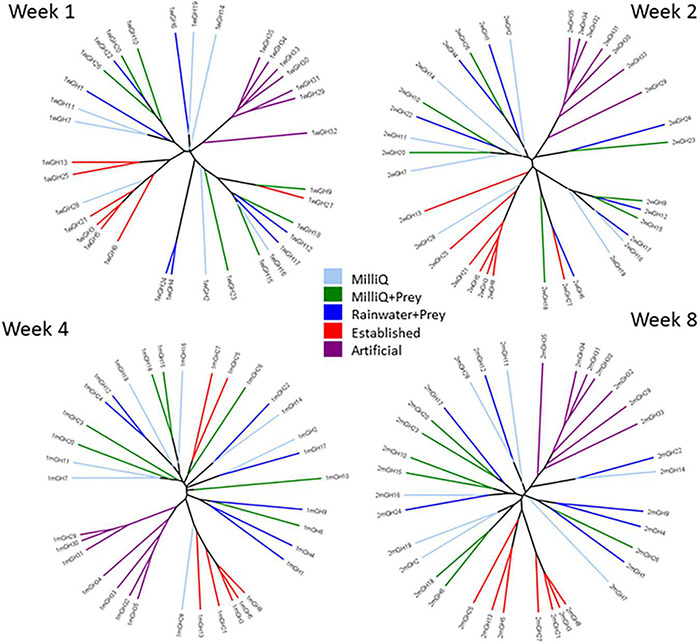
Sample relatedness Newick formatted UPGMA trees created using the Bray-Curtis index from composition of OTUs in all seven replicate pitchers at 4 time points—1, 2, 4, and 8 weeks. Branches are color-coded by treatment (treatment abbreviations the same as Figure 1). Individual pitchers across the 5 treatments are labeled as GH1–GH35.

In sample relatedness trees based on bacterial composition, individual pitcher samples showed clustering within treatments and the seven replicate pitchers for each treatment became more closely grouped together over time (Figure 4). For example, EST treatments were more closely clustered at week 8 than in earlier timepoints. EST and ART pitchers were the most distinct in bacterial composition with ART pitchers clustering separately from other treatment at all timepoints. MQ, MQP, and RWP pitchers were more scattered within the tree at week 1 and did not form distinct treatment clusters even by week 8.

### Core Microbiome and Full Bacterial Composition

**FIGURE 5:**
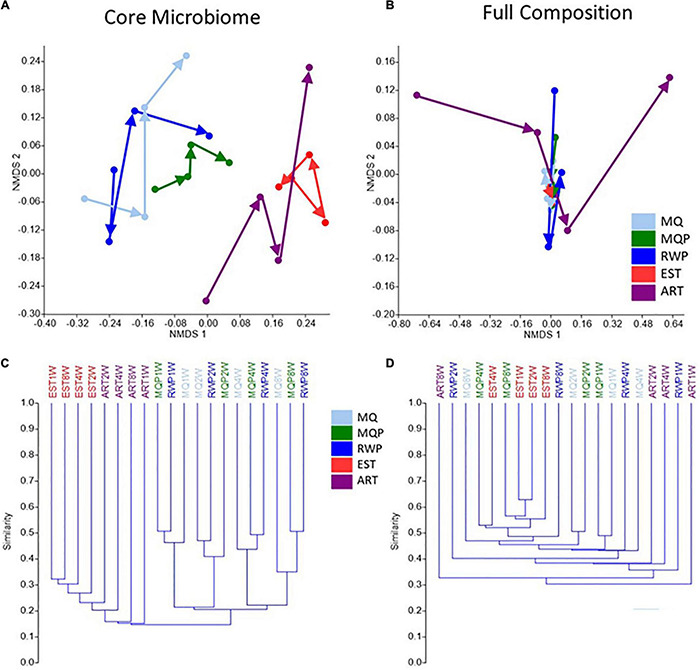
Pitcher bacterial composition for each of the five treatments, over time visualized with NMDS **(A,B)** and UPMGA trees **(C,D)** using Jaccard similarity index, comparing core microbiome **(A,C)** with full bacterial composition **(B,D)**. Each datapoint represents the pooled OTUs identified in 7 pitchers per treatment shows a timepoint (1, 2, 4, 8 weeks) and arrows indicate successional trajectories for the 5 treatments. Core microbiome was the bacterial OTUs present in at least 85% of all 7 replicate pitchers within each treatment. Treatment abbreviations are the same as Figure 1.

The core microbiome represents OTUs that are present in 85% of replicate pitchers within a given treatment and timepoint, whereas the full composition includes all OTUs (excluding singletons, chloroplast, mitochondria, and Archaeal taxa). 681 OTUs contributed to the core microbiome across all treatments vs. 22,367 total OTUs in the full composition dataset. Visualization of composition using NMDS and UMPGA trees showed notable treatment distinctions and clustering of timepoints, between core microbiome and full bacterial composition (Figure 5). The core microbiome NMDS showed clearer compositional trajectories with treatment, and tighter clustering of treatment timepoints, especially for ART and EST treatments than the full composition. In the UMPGA trees, the treatments in the core microbiome comparison were separated by lower similarity score than the full composition (Figures 5C,D). The core microbiome in the EST treatment was significantly different to all other treatments (ANOSIM *p* < 0.035) but EST was only significantly different to RWP in the full composition (*p* < 0.028). In core microbiome composition, ART was different from all living pitchers (*p* < 0.035) and ART was different to MQ (*p* < 0.026) and MQP (*p* < 0.031) in the full composition. The MQ, MQP, and RWP treatments in both full and core microbiome were closer in composition with no distinct clustering between timepoints.

### Cell Abundance

**FIGURE 6:**
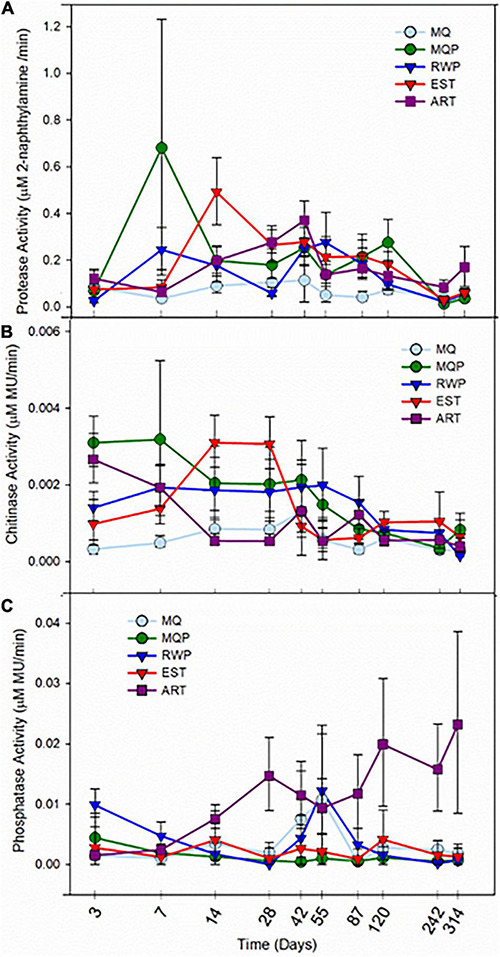
Activity of hydrolytic enzymes protease **(A)**, chitinase **(B)**, and alkaline phosphatase **(C)** in pitcher fluid within five treatments (MQ, MQP, RWP, EST, and ART) sampled over 314 days from the start of the greenhouse experiment. Treatment abbreviations are the same as for Figure 1. Points are means of values for seven replicate pitchers for each treatment and bars are standard error. The time axis is on a log scale.

Bacterial cell abundance (Supplementary Figure 3) showed some similar patterns over time to hydrolytic enzyme activities (Figure 6). The cell abundance in all treatments increased from day 3 onward (*p* < 0.045) except for day 55. EST pitchers had higher cell density than the assembling pitchers on day 14 (*p* < 0.008) and day 121 (*p* < 0.007).

### Enzyme Activity

Measurements of processing functions within the community, as protease, chitinase, and phosphatase activity (Figure 6) showed highest activities during the first few weeks after pitcher opening, coinciding with the highest cell abundances (Supplementary Figure 3), but protease and chitinase activity peaks occurred at different times between day 7 and 42, across treatments. In living pitchers, activity of enzymes declined to low levels at the end of the experiment when pitchers were senescing. EST pitchers showed relatively high but not always the highest activities, while activity in MQ only pitchers remained low. For both protease and chitinase there was significantly higher activity in MQP than MQ pitchers (*p* < 0.045, *p* < 0.0038, respectively, Supplementary Table 3). ART pitchers showed significantly higher chitinase activity than living pitcher treatments on day 7 (*p* < 0.0345) and day 42 (*p* < 0.025). The highest chitinase activity was in EST and MQP treatments and the lowest was in MQ pitchers (Figure 6B). Phosphatase activity (Figure 6C) was the most variable and higher activity was measured in ART pitchers than in living assembling communities (*p* < 0.0028). In ART pitchers, phosphatase activity continued to rise over time resulting in higher activity in ART pitchers than living pitcher treatments on day 87 (*p* < 0.036). RWP pitchers had higher phosphatase activity than the MQP treatment on day 3 (*p* < 0.002), day 7 (*p* < 0.008), and day 14 (*p* < 0.005).

### Nutrient Transformation

**FIGURE 7:**
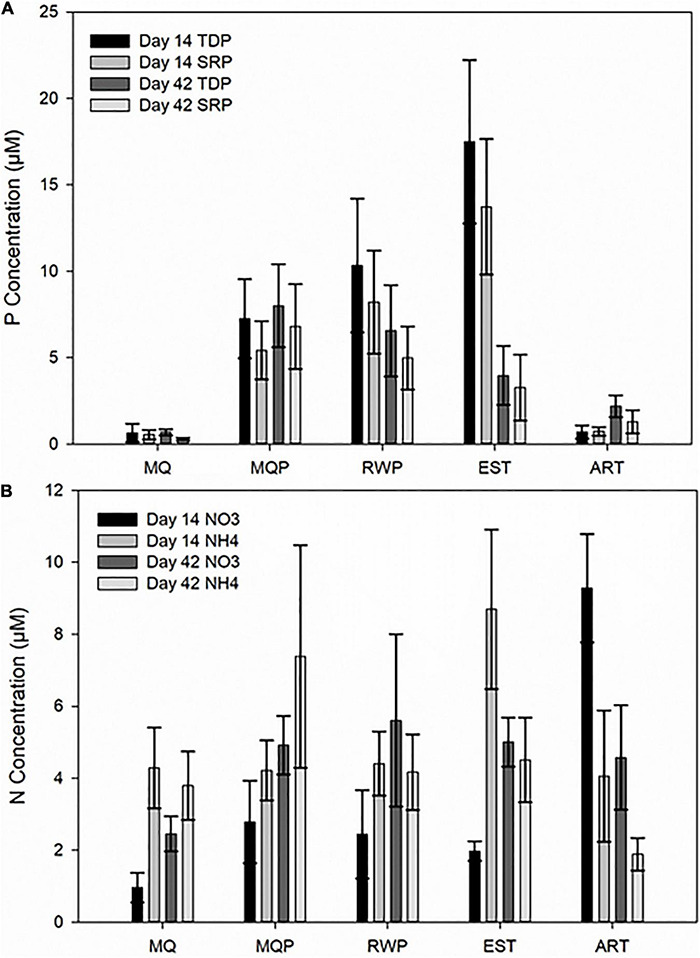
Dissolved nutrient concentrations measured in pitcher fluid samples collected on day 14 and 42. **(A)** Total dissolved phosphorus (TDP), soluble molybdate-reactive P (SRP), and **(B)** nitrate (NO3) and ammonium (NH4) concentrations. Treatment abbreviations are the same as Figure 1.

Both total dissolved P (TDP) and SRP concentrations remained very low in MQ without prey pitchers but dissolved P was also low in ART pitchers on day 14 and 42 (Figure 7). On day 14, EST pitchers had significantly higher TDP than other living pitcher treatments (*p* < 0.0001). MQP had higher TDP than MQ pitchers on day 14 (*p* < 0.01) and ART pitchers had lower TDP than other assembling communities (MQ, MQP, RWP) (*p* < 0.005). SRP was higher in MQP than MQ on day 14 (*p* < 0.0165), and on day 14 and 42, SRP was higher in EST than assembling living communities (*p* < 0.001). Ammonium concentrations did not differ with treatment, but dissolved nitrate was higher in ART than living pitcher treatments (*p* < 0.0001).

### Predicted Community Functions

**FIGURE 8:**
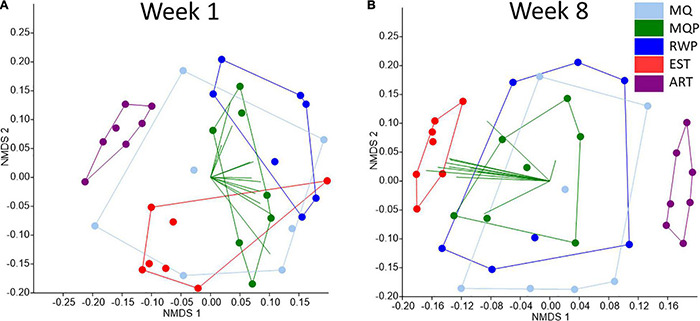
NMDS plots of predicted functional analysis of bacterial composition in 7 replicate pitchers in each of 5 treatments comparing week 1 **(A)** and week 8 **(B)**, using the Euclidean distance calculator. Both the NMDS data and the 12 vector overlays originate from PICRUSt-predicted bacterial metabolic functions. The 12 vectors included are nucleotides, kinase, Nitrogen, Signaling, Xenobiotics, Amino Acids, Arginine/Proline biosynthesis, Carbohydrate degradation, Bacterial Carbon fixation, Photosynthesis, Phenylalanine Tyrosine Tryptophan biosynthesis (PAA_TYR_TRP), and cell cycle and motility.

Functional predictions of community functions visualized with NMDS plots (weighted NSTI scores range of 0.020–0.273) showed tighter clustering of replicate pitchers within EST treatments and more scattered distributions at both timepoints in MQ, RWP, and MQP pitchers (Figure 8). By week 8, both EST and ART pitchers were separated from other treatments. In week 1, PICRUSt functional vectors were spread out amongst multiple treatments. In week 8, most functional vectors were aligned toward the EST pitcher communities, or within MQ, MQP, or RWP. The vector for photosynthesis traits was partially toward the artificial pitchers.

## Discussion

### Sources of Bacteria

During exposure to different bacterial sources, pitcher plant communities recruited diverse bacterial communities over the 8 weeks of composition monitoring. In assembling communities, bacterial richness was highest after just 2 weeks, despite later additions of new prey and a much longer life-span of the pitchers (∼1 year). The community analysis suggests that air, rainwater, and prey are all sources of bacteria for recruitment to the *S. purpurea* food web, but they are not equally influential. The contribution of bacteria from air in the greenhouse was common to all treatments, and there were few taxa present in other treatments but not found in milliQ only pitchers, suggesting a ubiquitous but significant bacterial recruitment from the air. In natural ecosystems, air above different land types can have an eightfold difference in bacterial abundance and can hold > 10^5^ bacteria-like particles per cubic meter (Bowers et al., 2011). Suspended water droplets may help disperse bacteria (Bowers et al., 2011). As new pitchers open, particles with attached bacteria can fall in, though differences between pitcher and surrounding wetland bacterial communities (Bittleston et al., 2018) suggest selection within the pitcher habitats. In milliQ only pitchers, lack of prey constrained nutrient resources and altered competition; the family *Simkaniaceae* which are known to thrive in oligotrophic conditions (Thao et al., 2003) were abundant in milliQ only, but may have been selected against in communities with greater prey nutrient resources.

Natural rainwater was not significant in recruiting a diverse bacterial community as the milliQ + prey, and rainwater + prey treatments were the very similar though *Spirosomaceae* showed more persistence in rainwater + prey than milliQ + prey pitchers. In this greenhouse experiment, rainwater was added at the beginning of the experiment, but in field plants, pitchers would typically receive regular rainfall which may contribute new bacteria. Heavy rainfall events can dilute communities, stimulating composition changes (Abbott and Battaglia, 2015). Rainwater contains up to 500 colony-forming bacteria per mL (Joly et al., 2013) with diversity including Proteobacteria, Actinobacteria, and Bacteriodetes (Kaushik et al., 2014), which is consistent with bacterial classes previously observed in North American pitcher plants (Koopman et al., 2010; Gray et al., 2012; Armitage, 2017; Canter et al., 2018; Grothjan and Young, 2019). While rainwater apparently contributed few unique taxa, warmer and wetter climates can produce larger pitchers (Ellison et al., 2004; Freedman et al., 2021) and this can affect food web composition and diversity (Ellison et al., 2004; Gotelli and Ellison, 2006; Krieger and Kourtev, 2012) so rainfall can also have an indirect effect on pitcher microbial communities.

Prey breakdown provides nutrients that support development of diverse communities. Addition of prey resulted in some families (*Neisseriaceae, Chtoniobacteraceae, Inquilinaceae, Moraxelleaceae*) increasing in abundance over time but remained in low abundance in milliQ only pitchers (Figure 2), suggesting contribution of these taxa from prey capture, or requirements for nutrients from prey breakdown. Insect prey surfaces and gut contents could contribute common taxa to pitchers; gut taxa found on Coleoptera, Diptera, Hemiptera, and Hymenoptera insect orders include Proteobacteria (Jones et al., 2013; Ramalho et al., 2017), and Rhizobiales, Xanthomonadales, and Burholderiales in guts of ants (*Cephalotes varians*) (Kautz et al., 2013), all bacterial taxa present in *S. purpurea* pitchers. Under natural conditions, catching different prey will likely contribute different suites of bacteria to each individual pitcher (Bradshaw and Creelman, 1984; Siragusa et al., 2007). Outside surfaces of pitchers or rooting substrate may have a minor influence on bacterial recruitment (Luciano and Newell, 2017). However, this study suggests that rain and air sources may contribute to bacterial recruitment in *S. purpurea*, and wetland habitat differences (Grothjan and Young, 2019) may provide different rain and air sources.

In contrast to temporal changes in communities starting *without* established communities, the tighter clustering of replicate pitchers in the established treatment, and the clear separation from the other treatments throughout the experiment, and the more stable temporal dynamics of community change over 8 weeks indicates that the “mature” food web and microbial communities in field pitchers provide stability to bacterial composition. This may relate to the higher bacterial richness and diversity in the established pitcher communities (Figure 3; McCann, 2000; Miller and TerHorst, 2012). The differences between the established community and other treatments also illustrates the idea of “historical contingency” in which the starting point for the microbial community composition influences successional changes over time (Bittleston et al., 2020), although the *Sarracenia* eukaryotic community composition has also been shown to homogenize over a growing season (Miller and TerHorst, 2012). The established field pitcher fluid was filtered (153 μm mesh) to exclude larger organisms, but ciliates, protozoa, and other smaller eukaryotes were likely included. So, in addition to bacteria added, the eukaryotes in the established “inoculum” likely influenced the bacterial communities as these are closely related (Bittleston et al., 2018; Grothjan and Young, 2019) and specific eukaryotes can influence bacterial diversity (Butler et al., 2008; Gray et al., 2012; Baiser et al., 2013; Canter et al., 2018). From a more diverse starting point than the assembling communities, the established communities lost richness and diversity over time when separated from the field environment (Figure 3), and this may be related to lack of the full eukaryotic food web composition. Established treatment pitchers also had the most unique low abundance taxa of any treatment suggesting recruitment of unique bacterial taxa from the wetland habitat which were not available in the greenhouse.

In the established treatment pitchers, eukaryotic and bacterial components of the mature communities were added simultaneously at the start of the experiment, whereas in natural ecosystems, recruitment of bacteria and pitcher colonization by eukaryotic food web components occurs over a few weeks after pitcher opening (Cresswell, 1991; Miller et al., 2002). In natural field pitchers abundant and taxonomically diverse bacteria have also been observed within 48 h of pitcher opening (Peeples and Kourtev, 2013).

### Host Plant Effects on Bacterial Community

Recent studies using plastic or glass tubes as “artificial” pitchers suggest some effect of the host tissue (Koopman et al., 2010; Bittleston et al., 2018; Ellison et al., 2021). In this study, the distinct differences in bacterial composition and successional trajectories in the artificial vs. live pitchers also supports the idea that the role of the host pitcher tissue is more than just an inert receptacle. Tight clustering and thus low variability between the seven replicate artificial pitchers (Figure 4) also suggested more similar conditions within the artificial ecosystems than in real pitchers. The host plant can affect communities by taking up nutrients released within the community and providing oxygen, as well as producing structures and nectar to attract insect prey (Wakefield et al., 2005; Bennett and Ellison, 2009; Adlassnig et al., 2011). Absence of host nutrient uptake could have resulted in the abundant algal growth in artificial pitchers, as algae are typically of low abundance in living pitchers (Gebühr et al., 2006; Young et al., 2018). Many of the highly abundant bacterial taxa in living pitchers were completely absent or minimally abundant in artificial pitchers (Figure 2). Other taxa, rare in artificial pitchers but common in real pitchers (*Microbacteriaceae, Acidobacteriaceae*) may have plant-specific functions as plant pathogens (Rakhashiya et al., 2015), and in cellulose degradation (Campbell, 2014) thus are absent from artificial pitchers. Other common freshwater taxa *Aeromonadaceae* and *Azospirillaceae* (Janda and Abbott, 2010; Baron et al., 2017) were common in artificial pitchers, but the absence of *Simkaniaceae* may be because of low nutrient requirements (Thao et al., 2003; Everett, 2014).

The plant tissue may provide a stable environment for microbial growth (Heard, 1994; Bittleston et al., 2018; Ellison et al., 2021) as well as contributing some early hydrolytic enzyme production (Gallie and Chang, 1997; Krieger and Kourtev, 2012). Nectar produced at the top of the pitcher to attract insects (Bennett and Ellison, 2009) could also be washed into the pitcher, contributing organic carbon. Natural pitchers may also emit volatile compounds for attracting prey or commensal mosquitoes (Heard, 1994; Ellison et al., 2021). Another study suggested that the host can impose some selection on bacteria, independent of prey capture (Bittleston et al., 2018) but the selective mechanisms are unknown. Known changes in the pitcher communities as pitchers age (Fish and Hall, 1978; Cipollini et al., 1994) also suggest some influence of the host pitcher status on the microbial communities (Ellison et al., 2021). Recruitment of microbes by host plants depends on the environment and plant location. In the rhizosphere, bacterial recruitment is influenced by root exudates (Kawasaki et al., 2016) and extensive selection of microbiome bacteria including endophytic bacteria occurs, with abiotic factors and plant defense systems interacting to determine microbiome composition (Jones et al., 2019). Endophytic microbes can be packaged with seeds to support beneficial colonization for the next generation (Shade et al., 2017). The variable composition of the microbial taxa across the treatments and replicate pitchers suggests both host pitcher tissue and bacteria sources influence the microbial communities. In *Sarracenia*, clearly the pitcher provides habitat and promotes prey capture but we currently lack evidence for more direct biochemical “management” of microbiomes by pitcher plants as has been demonstrated in other plants (Jones et al., 2019).

### Core Microbiome

The pitcher microbial composition can be examined using genetic sequencing to identify all the taxa present across replicate pitchers for each treatment. However, in terms of the most functionally important taxa, the concept of core microbiome may be useful, including the bacterial taxa most consistently found within a community (Hernandez-Agreda et al., 2017). This core microbiome should include bacterial taxa which are most consistently recruited and persist in pitchers, and thus may be of particular functional importance to the community or ecosystem (Baldrian et al., 2012; Chen et al., 2020; Liang et al., 2020). Across microbiome studies, what constitutes the core microbiome varies, with taxa present in 30–95% of samples (Huse et al., 2012; Ainsworth et al., 2015). However, biological justifications of the criteria are rare (Risely, 2020). Our definition of 85% or 6 of the 7 replicate pitchers in each treatment, leans to the more stringent side (Björk et al., 2017). Relatively few OTUs were found in all 7 pitchers at all timepoints but these included genera *Mucilaginibacter*, *Duganella, Curvibacter, Novosphingobium, Delftia, Pedobacter*, and *Aquitalea* which have all been reported in field samples from *S. purpurea* (Paisie et al., 2014; Grothjan and Young, 2019; Bittleston et al., 2020; Freedman et al., 2021) suggesting they may be functionally important in pitcher plant communities. Of these taxa, *Novosphingobium* and *Delftia*, along with *Simkaniaceae*, were part of core microbiomes in living pitcher treatments, i.e., found in at least 6 of the 7 replicate pitchers for each living pitcher treatment, but also absent from all replicate artificial pitchers. The clearer successional trajectories and clustering between treatments in core microbiome composition and higher variability in full composition (Figure 5) suggests that this core microbiome has tighter regulation, selection, or response to the environmental conditions within the pitchers and suggest that this core group of taxa more clearly define community responses to different conditions. The higher similarity between established pitcher timepoints also suggests that the more mature, taxonomically diverse communities are more stable over time than the assembling communities. This core microbiome analysis may provide a valuable approach to avoid compositional “noise” of possibly incidental taxa in environmental samples and focus on a core group of functionally important taxa. Understanding the core microbiome development is important for host plants. In human hosts, core microbiomes may share functions related to disease protection, and use of assembled artificial core microbiomes has been suggested for stabilizing human microbiomes and for increasing agricultural crop yield (Gopal et al., 2013; Hoisington et al., 2015).

### Microbiome Functions—Hydrolytic Enzymes

Bacteria perform essential functions within the food web and for the plant host by digesting organic prey particles into simpler and inorganic nutrient sources, chiefly by extracellular hydrolytic enzyme activities (Young et al., 2018). We can link bacterial composition to this enzyme activity. For example, *Chitinophagaceae* were common across most treatments, and this taxon produces chitinase activity critical for insect exoskeleton breakdown, though other taxa also produce chitinases (Young et al., 2018). Chitinase activity within the microbial community is stimulated by insect prey addition (Young et al., 2018) and the lower chitinase activity in pitchers without prey additions (therefore chitin substrate) supports this. Some elevated chitinase activity could relate to higher bacterial abundance (Supplementary Figure 3) as cells grow when prey resources are present; high chitinase activity in milliQ + prey pitchers on days 3 and 7 coincided with higher bacterial abundance. *S. purpurea* pitchers fed with ants, spiders, and other invertebrate prey, showed increases in microbial abundance over 15 days (Miller et al., 2002). Variable chitinase activity early in the experiment chitinase production in response to prey additions and some activity can also be released from prey during degradation (Young et al., 2018), as insects produce their own chitinases and proteases (Sharma et al., 1984; Kramer and Muthukrishnan, 1997). Bacterial prey digestion also depends on shredding by larger invertebrates (Cochran-Stafira and von Ende, 1998; Kneitel and Miller, 2002) but these were not included in this experiment. In the established community, chitinase activity peaked later than other treatments. Dissolved or fine particulate matter from field pitchers may have provided some nutrient resources, reducing the need for fast activation of chitinase seen in the other treatments when new insect prey was added.

Protease activity also responded to treatment. Within a few days of opening, some minor protease activity can be contributed by the plant (Gallie and Chang, 1997) in contrast to *Nepenthes* pitcher plants which actively excrete hydrolytic enzymes (Bačovčinova et al., 2018). For chitinase and protease, the early stimulation of activity followed by a slow decline over several weeks (Figure 6) is consistent with changes over time in field pitchers (Grothjan, 2021). In contrast, phosphatase activity increased over time in artificial pitchers, probably related to the growth of green algae which produce abundant extracellular phosphatase activity (Young et al., 2010; Vrba et al., 2018). *Aeromonadaceae*, which were only observed in high abundance in artificial pitchers (Figure 2) are also known for producing alkaline phosphatase (Ghosh and Homechaudhuri, 2011).

### Nutrient Transformations

Although bacterial transformation functions of prey nutrients have been known to be critical to the host plant and the food web, there have been few measurements of nutrient availability in these communities. Previously, we used scaled-down nutrient analysis techniques to show transformation of P sources within pitchers (Young et al., 2018) and this is the first analysis of both N and P transformation in pitcher communities. The host plants growing in the Cedarburg Bog have been shown to be N rather than P limited (Bott et al., 2008). Prey addition clearly added P to the communities (Figure 7) as P remained very low in milliQ only pitchers. The decline in TDP from days 14 to 42 suggests conversion to SRP and/or uptake by plant and microbial communities, indicating P release from prey. The low P in artificial pitchers was likely due to rapid hydrolysis of TDP into SRP by high phosphatase activity and rapid SRP uptake by abundant algae. Nitrate and ammonium measured in pitchers reflects degradation of organic N within insect prey. The pitcher plant host can take up these inorganic forms, and possibly some amino acids (Karagatzides et al., 2009) and bacteria could take up both inorganic and organic N forms, while algae abundant in artificial pitchers prefer inorganic ammonium or nitrate. In all living pitchers, a trend of higher nitrate on day 42, contrasted with artificial pitchers, where a decline in nitrate from days 14 to 42 may be related to algal uptake. Differences in living vs. artificial treatments suggest that the host plant may influence microbial communities via macronutrient uptake. Higher TDP (day 14) and phosphatase activity (days 3, 7, 14) in RWP than MQP suggests rainwater may have contributed some nutrients and conditions for phosphatase activity. Microbial functions in the pitchers clearly contribute to macronutrient cycling in the pitcher plant community.

### Community Functional Predictions

The 16S rRNA gene-taxonomy based bacteria functional predictions from PICRUSt can relate to traits involved in ecological functions of the pitcher plant microbial community (Bittleston et al., 2021) and showed changes between weeks 1 and 8 (Figure 8). The overlap of treatments at week 1 suggests more similar functions within communities. By week 8 the tighter clustering of established community functions suggests that the more diverse starting community resulted in more constrained community functions, possibly resulting from selection of functionally important taxa for nutrient transformations. This may also reflect the known importance of pitcher plant community age in microbial diversity and functions (Miller and TerHorst, 2012; Armitage, 2017).

Artificial pitchers hosted high algal abundance and cyanobacterial OTUs and the PICRUSt functional vector for photosynthesis was partially toward artificial pitchers. Photosynthetic traits within a community are readily identified through PICRUSt (Koo et al., 2017). The distinct functions of artificial pitchers from week 1 to 8 reflects the distinct composition in response to different recruitment and selection conditions than in living pitchers. There was no clear evidence for a specific species or group of bacterial taxa in living pitchers that were selected for or must be included in communities to achieve prey digestion, especially early in pitcher lifespans. Rather, diverse bacterial taxa may contribute similar functions to the *S. purpurea* microbial community, in terms of digestion and nutrient transformations (Young et al., 2018). This functional redundancy has been examined within microbial communities (Royalty and Steen, 2021) with some evidence that the environmental conditions select for which taxa perform the function (Tringe et al., 2005; Nunan et al., 2017). More detailed analyses of taxa and diversity-function relationships are needed to understand the diversity-functional dynamics during succession in microbial communities hosted by *S. purpurea*.

## Conclusion

Bacteria introduced into the *Sarracenia* food web come from a range of sources, although many abundant and ubiquitous taxa across all treatments suggest significant bacterial recruitment from the air, while rainwater was less important. Added insect prey contribute commonly found bacteria and provide nutrient resources that likely influences selection during bacterial community development. High bacterial diversity and food web eukaryotes in natural field pitchers may help to stabilize the bacterial community diversity. The host plant pitcher tissue also clearly influences microbial composition, partly through uptake of N and P macronutrients. Recruitment of bacteria from different sources also influences community functional capabilities. Hydrolytic enzyme activity, as a critical microbial function to transform organic nutrients, is stimulated by insect prey availability. This study broadly suggests that bacterial composition in host pitcher plants is related to both stochastic and specific bacterial recruitment and that the plant tissues influence selection within the microbiome, along with supporting food web assemblage through capture of insect prey.

## Data Availability Statement

The original contributions presented in the study are included in the article/Supplementary Material, further inquiries can be directed to the corresponding author/s.

## Author Contributions

JG and EY conceived and designed the experiments, performed the experiments, analyzed the data, contributed to reagents, materials, and analysis tools, prepared figures and tables, authored, reviewed drafts of the manuscript, approved the final draft, and collected samples. Both authors contributed to the article and approved the submitted version.

## Conflict of Interest

The authors declare that the research was conducted in the absence of any commercial or financial relationships that could be construed as a potential conflict of interest.

## Publisher’s Note

All claims expressed in this article are solely those of the authors and do not necessarily represent those of their affiliated organizations, or those of the publisher, the editors and the reviewers. Any product that may be evaluated in this article, or claim that may be made by its manufacturer, is not guaranteed or endorsed by the publisher.
