# Supplementary material for: Bacterial Recruitment to Carnivorous Pitcher Plant Communities: Identifying Sources Influencing Plant Microbiome Composition and Function
Source: Front Microbiol. 2022 Mar 14;13:791079. doi: 10.3389/fmicb.2022.791079 (PMC8964293; doi:10.3389/fmicb.2022.791079)
Supplement: Supplementary file 1 [file Data_Sheet_1.docx]

**Supplementary Materials**

**Materials and Methods**

*Plant selection, transplanting and greenhouse conditions*

Using a UWM Field station committee approved protocol, 15 plants were selected with the following criteria: 1) evident current season new growth 2) no crown senescence 3) a minimum of five healthy pitchers on the plant. In April 2015, plants were dug up carefully to include an undisturbed soil volume around each root system, transported to the UWM greenhouse, and potted into 10-inch plastic pots (sterilized by 70% ethanol, and rinsed with milliQ water three times). Plants were potted into sphagnum which had been heated at 60°C for 3 days then rehydrated in sterile milliQ water for 24 hours.

Plants were acclimated to transplantation and greenhouse conditions for 2 months, and to check for insect pests (including *Papaipema appassionata* moth larvae) that could disturb the experiment. Greenhouse conditions were maintained with a temperature range of 15-21^o^C and 65% humidity. To simulate winter dormancy, autumn conditions were started in November with 13-15^o^C and 65% humidity during the day and 55% at night. Spring conditions started in March, with an increase of temperature range to 2-16^o^C, simulating larger daily environmental temperature range. Following the initial transplant from field to greenhouse, irradiance was reduced to minimize stress, then in summer months irradiance was 650-700 μmol photons/m^2^/s but decreased during autumn and winter to maximum irradiance of 190-270 μmol photons/m^2^/s. Plants were watered daily with milliQ water to maintain damp sphagnum around the roots.

*Determination of Cell abundance*

Samples for epifluorescence measurement of bacterial density were filtered through 80 µm mesh and preserved with a final concentration of 1% glutaraldehyde (EM grade PolySciences Inc.) and stored at 4°C before staining with SYBR-Green, filtration and counting (Suttle and Fuhrman 2010) but with 0.2-μm black polycarbonate filters (Young, Sielicki & Grothjan, 2018).

**Table S1.** Sample volume (mL) removed from pitchers and parameters measured. Stars (*) indicate sampling dates where prey was added after sampling. Sterile MQ water was added to replace volume removed.

| Day of experiment (date starting in 2015) | Nutrient concentrations (mL) | Bacterial Counts (mL) | Enzyme activity (mL) | DNA extraction (mL) | Total volume (mL) |
| --- | --- | --- | --- | --- | --- |
| Day 0* (8/7) | -- | 1 | 1.2 | -- | 2.2 |
| Day 3 (8/10) | -- | 1 | 1.2 | -- | 2.2 |
| Day 7* (8/14) | -- | 1 | 1.2 | 5 | 7.2 |
| Day 14 (8/21) | -- | 1 | 1.2 | 5 | 7.2 |
| Day 28* (9/4) | -- | 1 | 1.2 | 5 | 7.2 |
| Day 42 (9/18) | 7.5 | 1 | 1.2 | 5 | 14.7 |
| Day 55* (10/1) | 7.5 | 1 | 1.2 | 5 | 14.7 |
| Day 88 (11/2) | 7.5 | 1 | 1.2 | 5 | 14.7 |
| Day 119 (12/4) | 7.5 | 1 | 1.2 | 5 | 14.7 |
| Day 242 (4/5) | 7.5 | 1 | 1.2 | 5 | 14.7 |
| Day 314 (6/16) | 7.5 | 1 | 1.2 | 5 | 14.7 |

**Table S2**. Summary of community composition parameters for bacterial composition in greenhouse experiments. Data are mean values (standard deviation) over 7 replicate pitchers per treatment for 5 treatments, MilliQ (MQ), MilliQ+Prey (MQP), Rainwater+Prey (RWP), Established community (EST), and Artificial pitchers (ART) at 4 timepoints. Statistical comparisons of Observed OTUs and Shannon Diversity index are presented in Table S3.

Supplementary Figures

**
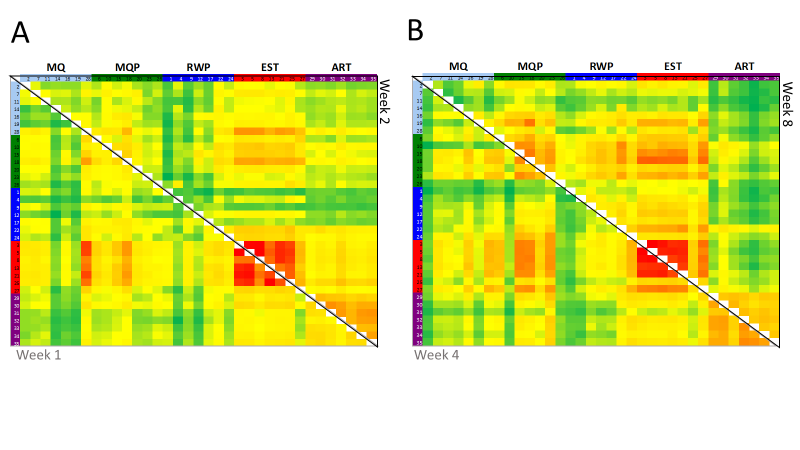
**

**Figure S1.** Heatmap matrix of composition comparisons showing similarity between samples for the 7 replicate pitchers in each treatment.Lower composition similarity between samples in green and higher similarity in red. Treatment colors and number of replicants are the same as in previous figures. **A**.Comparisons of samples between week 1 (lower triangle in grey) and week 2 (upper triangle in black). **B.**Comparison of samples between week 4 (lower triangle in grey) and week 8 (upper triangle in black).

**
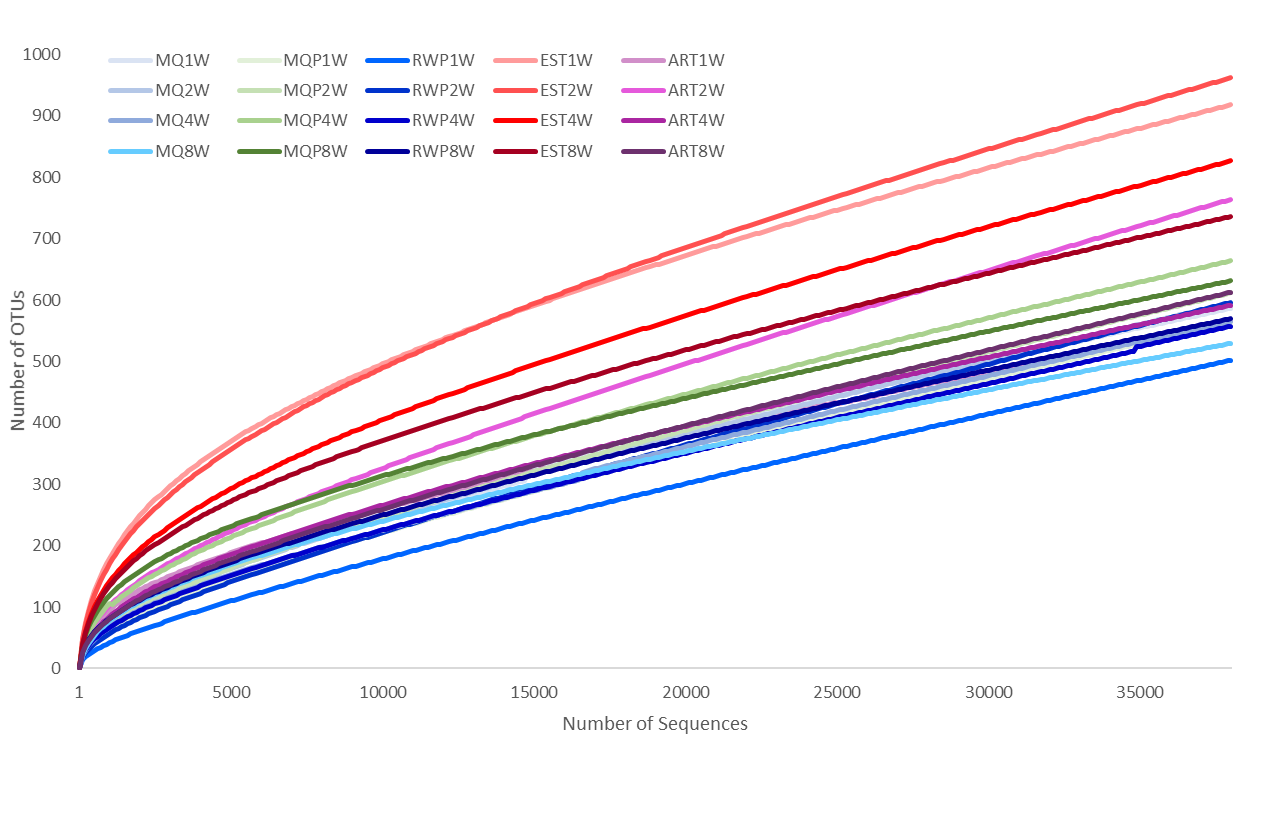
**

**Figure S2**. Rarefaction curves for OTUs vs sequencing depth for 16S RNA gene sequencing.Rarefaction curves showing OTUs identified in the 5 treatments (MQ, MQP, RWP, EST, and ART) and all timepoints (1W, 2W, 4W, 8W).

**Figure S3.**Bacterial cell abundance in 5 treatments (MQ, MQP, RWP, EST, and ART) over 8 timepoints. Points are mean values from 7 replicate pitchers per treatment and error bars are standard error. Treatment abbreviations are the same as Fig. 1. Time on the x-axis is plotted on a log scale.EST pitchers showed higher cell abundance on day 14, 28 and 119 than MQ (p < 0.016, 0.035, 0.002) and higher than ART pitchers on day 14 and 119 (p < 0.012, 0.003).

**Supplementary Table S3**. Statistic tables from lme models have orthogonal contrasts which includes the comparisons: 1) “MQP vs RWP” treatments to test for the effect of water source. 2) “MQ vs MQP” to test the effect of prey addition, 3) “ART vs PIT” where PIT is the four pooled living pitcher treatments (MQ, MQP, RWP), to test the effect of pitcher tissue against artificial (ART) treatment. 4) “EST vs AGE” to test the effect of a mature established community from the field (EST), against all the living pitcher assembling community treatments (MQ, MQP, RWP). Statistic are listed in the following order: diversity metrics (observed OTUs, Shannon diversity, singletons, Goods Coverage), enzymes (protease, chitinase, phosphatase), nutrients (TDP, SRP, NO3, NH4) and cell abundance. Models are separated by titles and "#" characters. A single row of "#" signs separate the next metric in a group while double rows of "#" characters separate groups of metrics.

---------------Observed OTUs----------------------

Model df AIC BIC LogLik Test L. Ratio p-value

lme.Obs.OTUs 1 4 1971.470 1983.237 -981.7351

lme.Obs.OTUs.Group 2 8 1936.499 1960.032 -960.2496 1 vs 2 42.97094 <.0001

lme.Obs.OTUs.Group.Time 3 11 1924.316 1956.674 -951.1580 2 vs3 18.18336 0.0004

#lme.Obs.OTUs.Group.Time.Inter 4 23 1917.818 1985.476 -935.9090 3 vs 4 30.49788 0.0023

Fixed effects: Obs..OTUs ~ Group + Timepoint + Group:Timepoint

Value Std.Error DF t-value p-value

(Intercept) 922.3071 18.91826 90 48.75222 0.0000

GroupMQPvsRWP 14.8929 34.53986 30 0.43118 0.6694

GroupMQvsMQP 93.6786 34.53986 30 2.71219 0.0110

GroupARTvsPIT -4.0786 12.61217 30 -0.32338 0.7486

GroupESTvsAGE 100.6714 12.61217 30 7.98208 0.0000

Timepoint.2W 36.2179 34.56857 90 1.04771 0.2976

Timepoint.4W -91.9571 34.56857 90 -2.66014 0.0092

Timepoint.8W 126.9001 34.56857 90 3.67097 0.0004

GroupMQPvsRWP:Timepoint.2W -16.6214 63.11328 90 -0.26336 0.7929

GroupMQvsMQP:Timepoint.2W -55.3800 63.11328 90 -0.87747 0.3826

GroupARTvsPIT:Timepoint.2W 66.3814 23.04571 90 2.88042 0.0050

GroupESTvsAGE:Timepoint.2W -45.2197 23.04571 90 -1.96217 0.0528

GroupMQPvsRWP:Timepoint.4W 91.9286 63.11328 90 1.45656 0.1487

GroupMQvsMQP:Timepoint.4W 153.9286 63.11328 90 2.43893 0.0167

GroupARTvsPIT:Timepoint.4W -69.4429 23.04571 90 -3.01327 0.0034

GroupESTvsAGE:Timepoint.4W -1.9429 23.04571 90 -0.08430 0.9330

GroupMQPvsRWP:Timepoint.8W 38.3645 63.11328 90 0.60787 0.5448

GroupMQvsMQP:Timepoint.8W 18.2399 63.11328 90 0.28900 0.7732

GroupARTvsPIT:Timepoint.8W 17.6053 23.04571 90 0.76393 0.4469

GroupESTvsAGE:Timepoint.8W -40.3940 23.04571 90 -1.75278 0.0830

#####################################################################################

-----------------------Shannon Diversity-----------------------------

Model df AIC BIC LogLik Test L. Ratio p-value

lme.Shannon 1 4 338.1157 349.8822 -165.0578

lme.Shannon.Group 2 8 313.8755 337.4087 -148.9378 1vs2 32.24013 <.0001

lme.Shannon.Group.Time 3 11 318.2246 350.5826 -148.1123 2vs3 1.65097 0.6479

lme.Shannon.Group.Time.Inter 4 23 307.8379 375.4956 -130.9189 3vs4 34.38671 0.0006

Fixed effects: Shannon ~ Group + Timepoint + Group:Timepoint

Value Std.Error DF t-value p-value

(Intercept) 2.9077531 0.07455126 90 39.00341 0.0000

GroupMQPvsRWP -0.2529908 0.13611136 30 -1.85870 0.0729

GroupMQvsMQP 0.1285955 0.13611136 30 0.94478 0.3523

GroupARTvsPIT -0.0035200 0.04970084 30 -0.07082 0.9440

GroupESTvsAGE 0.2944758 0.04970084 30 5.92497 0.0000

Timepoint.2W 0.0485918 0.10247235 90 0.47419 0.6365

Timepoint.4W 0.1261917 0.10247235 90 1.23147 0.2214

Timepoint.8W 0.0499485 0.10247235 90 0.48743 0.6271

GroupMQPvsRWP:Timepoint.2W 0.2787894 0.18708806 90 1.49015 0.1397

GroupMQvsMQP:Timepoint.2W 0.6051016 0.18708806 90 3.23431 0.0017

GroupARTvsPIT:Timepoint.2W -0.1397353 0.06831490 90 -2.04546 0.0437

GroupESTvsAGE:Timepoint.2W -0.2256366 0.06831490 90 -3.30289 0.0014

GroupMQPvsRWP:Timepoint.4W -0.0189035 0.18708806 90 -0.10104 0.9197

GroupMQvsMQP:Timepoint.4W 0.0814277 0.18708806 90 0.43524 0.6644

GroupARTvsPIT:Timepoint.4W 0.0329775 0.06831490 90 0.48273 0.6305

GroupESTvsAGE:Timepoint.4W -0.0273840 0.06831490 90 -0.40085 0.6895

GroupMQPvsRWP:Timepoint.8W 0.2335118 0.18708806 90 1.24814 0.2152

GroupMQvsMQP:Timepoint.8W 0.0724473 0.18708806 90 0.38724 0.6995

GroupARTvsPIT:Timepoint.8W 0.0832340 0.06831490 90 1.21839 0.2263

GroupESTvsAGE:Timepoint.8W 0.0429901 0.06831490 90 0.62929 0.5308

#####################################################################################

--------------------------Singletons------------------------------------------

Model df AIC BIC LogLik Test L. Ratio p-value

lme.Sing 1 4 1872.576 1884.342 -932.2879

lme.Sing.Group 2 8 1860.186 1883.719 -922.0931 1 vs 2 20.38968 4e-04

lme.Sing.Group.Time 3 11 1848.465 1880.823 -913.2326 2 vs 3 17.72084 5e-04

lme.Sing.Group.Time.Inter 4 23 1827.536 1895.193 -890.7678 3 vs 4 44.92962 <.0001

Fixed effects: Singletons ~ Group + Timepoint + Group:Timepoint

Value Std.Error DF t-value p-value

(Intercept) 662.3857 12.80637 90 51.72313 0.0000

GroupMQPvsRWP 40.1786 23.38113 30 1.71842 0.0960

GroupMQvsMQP 79.4643 23.38113 30 3.39865 0.0019

GroupARTvsPIT 4.0857 8.53758 30 0.47856 0.6357

GroupESTvsAGE 33.7643 8.53758 30 3.95478 0.0004

Timepoint.2W 21.3513 25.61275 90 0.83362 0.4067

Timepoint.4W -74.5143 25.61275 90 -2.90927 0.0046

Timepoint.8W 92.9182 25.61275 90 3.62781 0.0005

GroupMQPvsRWP:Timepoint.2W -29.0369 46.76226 90 -0.62095 0.5362

GroupMQvsMQP:Timepoint.2W -98.6745 46.76226 90 -2.11013 0.0376

GroupARTvsPIT:Timepoint.2W 62.7334 17.07516 90 3.67396 0.0004

GroupESTvsAGE:Timepoint.2W 1.3906 17.07516 90 0.08144 0.9353

GroupMQPvsRWP:Timepoint.4W 87.6905 46.76226 90 1.87524 0.0640

GroupMQvsMQP:Timepoint.4W 143.8810 46.76226 90 3.07686 0.0028

GroupARTvsPIT:Timepoint.4W -48.6857 17.07516 90 -2.85126 0.0054

GroupESTvsAGE:Timepoint.4W -6.4238 17.07516 90 -0.37621 0.7076

GroupMQPvsRWP:Timepoint.8W 35.4683 46.76226 90 0.75848 0.4501

GroupMQvsMQP:Timepoint.8W 28.2277 46.76226 90 0.60364 0.5476

GroupARTvsPIT:Timepoint.8W 18.3272 17.07516 90 1.07333 0.2860

GroupESTvsAGE:Timepoint.8W -42.7600 17.07516 90 -2.50422 0.0141

#####################################################################################

-----------------------------Goods Coverage-------------------------------

Model df AIC BIC LogLik Test L. Ratio p-value

lme.Gcov 1 4 -1082.726 -1070.959 545.3630

lme.Gcov.Group 2 8 -1077.514 -1053.981 546.7572 1 vs 2 2.788298 0.5939

lme.Gcov.Group.Time 3 11 -1076.461 -1044.103 549.2308 2 vs 3 4.947132 0.1757

lme.Gcov.Group.Time.Inter 4 23 -1069.622 -1001.964 557.8109 3 vs 4 17.160186 0.1437

Fixed effects: Singletons ~ Group + Timepoint + Group:Timepoint

Value Std.Error DF t-value p-value

(Intercept) 0.9891064 0.0004230226 90 2338.1879 0.0000

GroupMQPvsRWP 0.0005570 0.0007723301 30 0.7212 0.4764

GroupMQvsMQP -0.0006826 0.0007723301 30 -0.8838 0.3838

GroupARTvsPIT -0.0000351 0.0002820151 30 -0.1245 0.9017

GroupESTvsAGE -0.0000622 0.0002820151 30 -0.2207 0.8268

Timepoint.2W 0.0007538 0.0008139963 90 0.9260 0.3569

Timepoint.4W 0.0015348 0.0008139963 90 1.8855 0.0626

Timepoint.8W 0.0006100 0.0008139963 90 0.7494 0.4556

GroupMQPvsRWP:Timepoint.2W 0.0002974 0.0014861471 90 0.2001 0.8419

GroupMQvsMQP:Timepoint.2W -0.0010397 0.0014861471 90 -0.6996 0.4860

GroupARTvsPIT:Timepoint.2W 0.0001570 0.0005426642 90 0.2893 0.7730

GroupESTvsAGE:Timepoint.2W 0.0000006 0.0005426642 90 0.0010 0.9992

GroupMQPvsRWP:Timepoint.4W -0.0009892 0.0014861471 90 -0.6656 0.5074

GroupMQvsMQP:Timepoint.4W 0.0017391 0.0014861471 90 1.1702 0.2450

GroupARTvsPIT:Timepoint.4W -0.0004862 0.0005426642 90 -0.8959 0.3727

GroupESTvsAGE:Timepoint.4W 0.0000300 0.0005426642 90 0.0552 0.9561

GroupMQPvsRWP:Timepoint.8W -0.0023660 0.0014861471 90 -1.5920 0.1149

GroupMQvsMQP:Timepoint.8W 0.0013629 0.0014861471 90 0.9171 0.3615

GroupARTvsPIT:Timepoint.8W -0.0009661 0.0005426642 90 -1.7803 0.0784

GroupESTvsAGE:Timepoint.8W -0.0002830 0.0005426642 90 -0.5215 0.6033

#############################################################################

#############################################################################

------------------------------------Protease-----------------------------------

Model df AIC BIC LogLik Test L. Ratio p-value lmeProt 1 4 57.91889 73.73186 -24.959445

lmeProt.Treat 2 8 57.88394 89.50989 -20.941972 1 vs 2 8.03494 0.0903

lmeProt.Treat.Time 3 18 38.66119 109.81957 -1.330594 2 vs 3 39.22276 <.0001

lmeProt.Treat.Time.Inter 4 58 59.98593 289.27404 28.007037 3 vs 4 58.67526 0.0286

Fixed effects: Protease ~ Treatment + Time + Treatment:Time

Value Std.Error DF t-value p-value

(Intercept) 0.14552736 0.01679784 300 8.663458 0.0000

TreatmentMQPvsRWP 0.00176117 0.03066852 30 0.057426 0.9546

TreatmentMQvsMQP 0.06480935 0.03066852 30 2.113221 0.0430

TreatmentARTvsPIT 0.00555419 0.01119856 30 0.495974 0.6235

TreatmentESTvsAGE 0.01274441 0.01119856 30 1.138040 0.2641

Time.3 -0.03166421 0.03984512 300 -0.794682 0.4274

Time.7 -0.22018149 0.03984512 300 -5.525933 0.0000

Time.14 0.07276983 0.03984512 300 1.826317 0.0688

Time^28 0.00670063 0.03984512 300 0.168167 0.8666

Time^42 0.01197619 0.03984512 300 0.300569 0.7640

Time^55 0.06697701 0.03984512 300 1.680934 0.0938

Time^87 -0.00757013 0.03984512 300 -0.189989 0.8494

Time^121 0.06387434 0.03984512 300 1.603066 0.1100

Time^242 0.01171046 0.03984512 300 0.293899 0.7690

Time^314 -0.05534642 0.03984512 300 -1.389039 0.1659

TreatmentMQPvsRWP:Time.3 0.04791013 0.07274691 300 0.658587 0.5107

TreatmentMQvsMQP:Time.3 -0.02459184 0.07274691 300 -0.338047 0.7356

TreatmentARTvsPIT:Time.3 0.03072490 0.02656341 300 1.156662 0.2483

TreatmentESTvsAGE:Time.3 -0.00721937 0.02656341 300 -0.271779 0.7860

TreatmentMQPvsRWP:Time.7 -0.04350827 0.07274691 300 -0.598077 0.5502

TreatmentMQvsMQP:Time.7 -0.11513403 0.07274691 300 -1.582666 0.1146

TreatmentARTvsPIT:Time.7 0.00734284 0.02656341 300 0.276427 0.7824

TreatmentESTvsAGE:Time.7 -0.02790603 0.02656341 300 -1.050544 0.2943

TreatmentMQPvsRWP:Time.14 -0.04804996 0.07274691 300 -0.660509 0.5094

TreatmentMQvsMQP:Time.14 0.04026064 0.07274691 300 0.553434 0.5804

TreatmentARTvsPIT:Time.14 0.00680198 0.02656341 300 0.256066 0.7981

TreatmentESTvsAGE:Time.14 -0.00008406 0.02656341 300 -0.003164 0.9975

TreatmentMQPvsRWP:Time^28 0.08628932 0.07274691 300 1.186158 0.2365

TreatmentMQvsMQP:Time^28 -0.07005965 0.07274691 300 -0.963060 0.3363

TreatmentARTvsPIT:Time^28 0.04095648 0.02656341 300 1.541838 0.1242

TreatmentESTvsAGE:Time^28 0.02667715 0.02656341 300 1.004282 0.3161

TreatmentMQPvsRWP:Time^28 0.04408709 0.07274691 300 0.606034 0.5450

TreatmentMQvsMQP:Time^42 0.04850930 0.07274691 300 0.666823 0.5054

TreatmentARTvsPIT:Time^42 -0.00591932 0.02656341 300 -0.222837 0.8238

TreatmentESTvsAGE:Time^42 -0.03546739 0.02656341 300 -1.335197 0.1828

TreatmentMQPvsRWP:Time^55 -0.02375182 0.07274691 300 -0.326499 0.7443

TreatmentMQvsMQP:Time^55 0.11887550 0.07274691 300 1.634097 0.1033

TreatmentARTvsPIT:Time^55 -0.04817260 0.02656341 300 -1.813494 0.0708

TreatmentESTvsAGE:Time^55 0.01946252 0.02656341 300 0.732682 0.4643

TreatmentMQPvsRWP:Time^87 -0.07109796 0.07274691 300 -0.977333 0.3292

TreatmentMQvsMQP:Time^87 -0.14629009 0.07274691 300 -2.010946 0.0452

TreatmentARTvsPIT:Time^87 0.04000724 0.02656341 300 1.506103 0.1331

TreatmentESTvsAGE:Time^87 0.03202618 0.02656341 300 1.205650 0.2289

TreatmentMQPvsRWP:Time^121 -0.06147657 0.07274691 300 -0.845075 0.3987

TreatmentMQvsMQP:Time^121 0.12250535 0.07274691 300 1.683994 0.0932

TreatmentARTvsPIT:Time^121 -0.01234889 0.02656341 300 -0.464883 0.6424

TreatmentESTvsAGE:Time^121 -0.05899792 0.02656341 300 -2.221022 0.0271

TreatmentMQPvsRWP:Time^242 0.07144054 0.07274691 300 0.982042 0.3269

TreatmentMQvsMQP:Time^242 -0.03989039 0.07274691 300 -0.548345 0.5839

TreatmentARTvsPIT:Time^242 -0.01010101 0.02656341 300 -0.380260 0.7040

TreatmentESTvsAGE:Time^242 0.04316034 0.02656341 300 1.624804 0.1053

TreatmentMQPvsRWP:Time^314 -0.02368213 0.07274691 300 -0.325541 0.7450

TreatmentMQvsMQP:Time^314 -0.00865071 0.07274691 300 -0.118915 0.9054

TreatmentARTvsPIT:Time^314 -0.01467982 0.02656341 300 -0.552633 0.5809

TreatmentESTvsAGE:Time^314 -0.01541605 0.02656341 300 -0.580349 0.5621

######################################################################################################################

------------------------------Chitinase--------------------------------------

Model df AIC BIC LogLik Test L. Ratio p-value lmeCht 1 4 -3862.263 -3846.450 1935.131

lmeCht.Treat 2 8 -3865.324 -3833.698 1940.662 1 vs 2 11.06127 0.0259

lmeCht.Treat.Time 3 18 -3886.511 -3815.353 1961.256 2 vs 3 41.18717 <.0001

lmeCht.Treat.Time.Inter 4 58 -3867.613 -3638.325 1991.807 3 vs 4 61.10180 0.0174

Fixed effects: Chitinase ~ Treatment + Time + Treatment:Time

Value Std.Error DF t-value p-value

(Intercept) 0.0012188597 0.0001069268 300 11.399009 0.0000

TreatmentMQPvsRWP 0.0002372186 0.0001952208 30 1.215130 0.2338

TreatmentMQvsMQP 0.0006136580 0.0001952208 30 3.143406 0.0037

TreatmentARTvsPIT -0.0000461351 0.0000712845 30 -0.647196 0.5224

TreatmentESTvsAGE 0.0000684926 0.0000712845 30 0.960834 0.3443

Time.3 -0.0012600430 0.0002416730 300 -5.213835 0.0000

Time.7 -0.0007928655 0.0002416730 300 -3.280737 0.0012

Time.14 0.0005567270 0.0002416730 300 2.303638 0.0219

Time^28 -0.0001615879 0.0002416730 300 -0.668622 0.5043

Time^42 0.0000479790 0.0002416730 300 0.198528 0.8428

Time^55 -0.0001115905 0.0002416730 300 -0.461742 0.6446

Time^87 0.0001241591 0.0002416730 300 0.513748 0.6078

Time^121 0.0000985027 0.0002416730 300 0.407587 0.6839

Time^242 -0.0000782627 0.0002416730 300 -0.323837 0.7463

Time^314 -0.0000843418 0.0002416730 300 -0.348991 0.7273

TreatmentMQPvsRWP:Time.3 -0.0001509013 0.0004412325 300 -0.342000 0.7326

TreatmentMQvsMQP:Time.3 -0.0009411675 0.0004412325 300 -2.133042 0.0337

TreatmentARTvsPIT:Time.3 -0.0000548736 0.0001611153 300 -0.340586 0.7337

TreatmentESTvsAGE:Time.3 -0.0000359678 0.0001611153 300 -0.223243 0.8235

TreatmentMQPvsRWP:Time.7 -0.0002509411 0.0004412325 300 -0.568728 0.5700

TreatmentMQvsMQP:Time.7 -0.0003720061 0.0004412325 300 -0.843107 0.3998

TreatmentARTvsPIT:Time.7 0.0003425231 0.0001611153 300 2.125950 0.0343

TreatmentESTvsAGE:Time.7 0.0000739823 0.0001611153 300 0.459188 0.6464

TreatmentMQPvsRWP:Time.14 -0.0006595710 0.0004412325 300 -1.494838 0.1360

TreatmentMQvsMQP:Time.14 0.0004393280 0.0004412325 300 0.995684 0.3202

TreatmentARTvsPIT:Time.14 -0.0001654035 0.0001611153 300 -1.026616 0.3054

TreatmentESTvsAGE:Time.14 0.0001335539 0.0001611153 300 0.828934 0.4078

TreatmentMQPvsRWP:Time^28 0.0003331862 0.0004412325 300 0.755126 0.4508

TreatmentMQvsMQP:Time^28 -0.0004214312 0.0004412325 300 -0.955123 0.3403

TreatmentARTvsPIT:Time^28 -0.0002294408 0.0001611153 300 -1.424078 0.1555

TreatmentESTvsAGE:Time^28 0.0001472140 0.0001611153 300 0.913718 0.3616

TreatmentMQPvsRWP:Time^42 -0.0001723665 0.0004412325 300 -0.390648 0.6963

TreatmentMQvsMQP:Time^42 0.0005299098 0.0004412325 300 1.200976 0.2307

TreatmentARTvsPIT:Time^42 0.0003683908 0.0001611153 300 2.286504 0.0229

TreatmentESTvsAGE:Time^42 -0.0006450259 0.0001611153 300 -4.003505 0.0001

TreatmentMQPvsRWP:Time^55 0.0002787223 0.0004412325 300 0.631690 0.5281

TreatmentMQvsMQP:Time^55 -0.0000711972 0.0004412325 300 -0.161360 0.8719

TreatmentARTvsPIT:Time^55 -0.0001582694 0.0001611153 300 -0.982336 0.3267

TreatmentESTvsAGE:Time^55 0.0002374558 0.0001611153 300 1.473825 0.1416

TreatmentMQPvsRWP:Time^87 -0.0003349073 0.0004412325 300 -0.759027 0.4484

TreatmentMQvsMQP:Time^87 -0.0002516901 0.0004412325 300 -0.570425 0.5688

TreatmentARTvsPIT:Time^87 -0.0000528896 0.0001611153 300 -0.328271 0.7429

TreatmentESTvsAGE:Time^87 0.0002250187 0.0001611153 300 1.396631 0.1636

TreatmentMQPvsRWP:Time^121 -0.0002974845 0.0004412325 300 -0.674213 0.5007

TreatmentMQvsMQP:Time^121 -0.0000530057 0.0004412325 300 -0.120131 0.9045

TreatmentARTvsPIT:Time^121 0.0000600140 0.0001611153 300 0.372491 0.7098

TreatmentESTvsAGE:Time^121 -0.0002628508 0.0001611153 300 -1.631445 0.1038

TreatmentMQPvsRWP:Time^242 -0.0000534330 0.0004412325 300 -0.121099 0.9037

TreatmentMQvsMQP:Time^242 -0.0001748392 0.0004412325 300 -0.396252 0.6922

TreatmentARTvsPIT:Time^242 -0.0001560096 0.0001611153 300 -0.968310 0.3337

TreatmentESTvsAGE:Time^242 0.0000360368 0.0001611153 300 0.223671 0.8232

TreatmentMQPvsRWP:Time^314 0.0001069513 0.0004412325 300 0.242392 0.8086

TreatmentMQvsMQP:Time^314 0.0001859881 0.0004412325 300 0.421519 0.6737

TreatmentARTvsPIT:Time^314 -0.0001584030 0.0001611153 300 -0.983166 0.3263

TreatmentESTvsAGE:Time^314 0.0001641495 0.0001611153 300 1.018832 0.3091

#############################################################################

-------------------------------------APA------------------------------------

Model df AIC BIC LogLik Test L. Ratio p-value

lmeAPA 1 4 -2369.398 -2353.585 1188.699

lmeAPA.Treat 2 8 -2374.285 -2342.660 1195.143 1 vs 2 12.88753 0.0118

lmeAPA.Treat.Time 3 18 -2368.177 -2297.018 1202.088 2 vs 3 13.89097 0.1780

lmeAPA.Treat.Time.Inter 4 58 -2377.404 -2148.115 1246.702 3 vs 4 89.22705 <.0001

Fixed effects: APA ~ Treatment + Time + Treatment:Time

Value Std.Error DF t-value p-value

(Intercept) 0.005249006 0.0009434158 300 5.563831 0.0000

TreatmentMQPvsRWP 0.002569123 0.0017224337 30 1.491566 0.1463

TreatmentMQvsMQP 0.000343831 0.0017224337 30 0.199619 0.8431

TreatmentARTvsPIT 0.002058924 0.0006289439 30 3.273622 0.0027

TreatmentESTvsAGE -0.000590535 0.0006289439 30 -0.938931 0.3553

Time.3 -0.000779980 0.0016338919 300 -0.477376 0.6334

Time.7 0.002291202 0.0016338919 300 1.402297 0.1619

Time.14 -0.003821182 0.0016338919 300 -2.338700 0.0200

Time^28 0.003573537 0.0016338919 300 2.187132 0.0295

Time^42 -0.000149846 0.0016338919 300 -0.091711 0.9270

Time^55 -0.000056953 0.0016338919 300 -0.034857 0.9722

Time^87 0.000038710 0.0016338919 300 0.023692 0.9811

Time^121 0.001016211 0.0016338919 300 0.621957 0.5344

Time^242 0.002576180 0.0016338919 300 1.576714 0.1159

Time^314 0.000956154 0.0016338919 300 0.585200 0.5589

TreatmentMQPvsRWP:Time.3 -0.009810006 0.0029830648 300 -3.288566 0.0011

TreatmentMQvsMQP:Time.3 -0.006089629 0.0029830648 300 -2.041400 0.0421

TreatmentARTvsPIT:Time.3 0.006847262 0.0010892613 300 6.286152 0.0000

TreatmentESTvsAGE:Time.3 -0.001523345 0.0010892613 300 -1.398512 0.1630

TreatmentMQPvsRWP:Time.7 0.008105145 0.0029830648 300 2.717053 0.0070

TreatmentMQvsMQP:Time..7 0.006964136 0.0029830648 300 2.334557 0.0202

TreatmentARTvsPIT:Time.7 -0.000252707 0.0010892613 300 -0.231999 0.8167

TreatmentESTvsAGE:Time.7 0.000819799 0.0010892613 300 0.752619 0.4523

TreatmentMQPvsRWP:Time.14 -0.008504512 0.0029830648 300 -2.850931 0.0047

TreatmentMQvsMQP:Time.14 -0.002980196 0.0029830648 300 -0.999038 0.3186

TreatmentARTvsPIT:Time.14 0.001679170 0.0010892613 300 1.541568 0.1242

TreatmentESTvsAGE:Time.14 -0.000416856 0.0010892613 300 -0.382696 0.7022

TreatmentMQPvsRWP:Time^28 0.004539336 0.0029830648 300 1.521702 0.1291

TreatmentMQvsMQP:Time^28 -0.000688794 0.0029830648 300 -0.230902 0.8175

TreatmentARTvsPIT:Time^28 0.000236193 0.0010892613 300 0.216838 0.8285

TreatmentESTvsAGE:Time^28 -0.000253611 0.0010892613 300 -0.232829 0.8161

TreatmentMQPvsRWP:Time^42 0.000666991 0.0029830648 300 0.223593 0.8232

TreatmentMQvsMQP:Time^42 0.000536887 0.0029830648 300 0.179978 0.8573

TreatmentARTvsPIT:Time^42 -0.001285406 0.0010892613 300 -1.180071 0.2389

TreatmentESTvsAGE:Time^42 -0.000910248 0.0010892613 300 -0.835657 0.4040

TreatmentMQPvsRWP:Time^55 0.000909203 0.0029830648 300 0.304788 0.7607

TreatmentMQvsMQP:Time^55 0.001462789 0.0029830648 300 0.490365 0.6242

TreatmentARTvsPIT:Time^55 0.000582575 0.0010892613 300 0.534835 0.5932

TreatmentESTvsAGE:Time^55 0.000606761 0.0010892613 300 0.557039 0.5779

TreatmentMQPvsRWP:Time^87 -0.002895224 0.0029830648 300 -0.970554 0.3326

TreatmentMQvsMQP:Time^87 0.000340729 0.0029830648 300 0.114221 0.9091

TreatmentARTvsPIT:Time^87 0.002295173 0.0010892613 300 2.107091 0.0359

TreatmentESTvsAGE:Time^87 0.000161475 0.0010892613 300 0.148243 0.8823

TreatmentMQPvsRWP:Time^121 -0.000276242 0.0029830648 300 -0.092603 0.9263

TreatmentMQvsMQP:Time^121 -0.001132147 0.0029830648 300 -0.379525 0.7046

TreatmentARTvsPIT:Time^121 0.000436800 0.0010892613 300 0.401006 0.6887

TreatmentESTvsAGE:Time^121 -0.000077688 0.0010892613 300 -0.071322 0.9432

TreatmentMQPvsRWP:Time^242 0.000614806 0.0029830648 300 0.206099 0.8369

TreatmentMQvsMQP:Time^242 -0.002208843 0.0029830648 300 -0.740461 0.4596

TreatmentARTvsPIT:Time^242 -0.000998802 0.0010892613 300 -0.916953 0.3599

TreatmentESTvsAGE:Time^242 0.000093883 0.0010892613 300 0.086189 0.9314

TreatmentMQPvsRWP:Time^314 0.001237865 0.0029830648 300 0.414964 0.6785

TreatmentMQvsMQP:Time^314 0.000220434 0.0029830648 300 0.073895 0.9411

TreatmentARTvsPIT:Time^314 0.000229407 0.0010892613 300 0.210608 0.8333

TreatmentESTvsAGE:Time^314 -0.000587462 0.0010892613 300 -0.539321 0.5901

#####################################################################################

#####################################################################################

--------------------------------------------TDP-----------------------------------

Model df AIC BIC LogLik Test L. Ratio p-value

lme.TDP 1 4 481.2470 490.1834 -236.6235

lme.TDP.Treat 2 8 472.7208 490.5936 -228.3604 1 vs 2 16.526186 0.0024

lme.TDP.Treat.Day 3 9 470.2508 490.3577 -226.1254 2 vs 3 4.470019 0.0345

lme.TDP.Treat.Day.Inter 4 13 454.5840 483.6274 -214.2920 3 vs 4 23.666777 0.0001

Fixed effects: TDP ~ Treatment + Day + Treatment:Day

Value Std.Error DF t-value p-value

(Intercept) 7.288000 1.0760895 30 6.772671 0.0000

TreatmentMQPvsRWP 4.249623 1.9646616 30 2.163031 0.0386

TreatmentMQvsMQP 5.425273 1.9646616 30 2.761429 0.0097

TreatmentARTvsPIT -2.195897 0.7173930 30 -3.060940 0.0046

TreatmentESTvsAGE 3.403386 0.7173930 30 4.744103 0.0000

Day42 -2.921711 1.0421893 29 -2.803436 0.0089

TreatmentMQPvsRWP:Day42 -2.498839 1.9576265 29 -1.276464 0.2119

TreatmentMQvsMQP:Day42 -0.888254 1.8987895 29 -0.467800 0.6434

TreatmentARTvsPIT:Day42 1.472171 0.6882304 29 2.139067 0.0410

TreatmentESTvsAGE:Day42 -3.533823 0.6882304 29 -5.134651 0.0000

#####################################################################################

-----------------------------SRP----------------------------

Model df AIC BIC LogLik Test L. Ratio p-value

lme.SRP 1 4 459.2792 468.2732 -225.6396

lme.SRP.Treat 2 8 451.9771 469.9651 -217.9886 1 vs 2 15.302075 0.0041

lme.SRP.Treat.Day 3 9 449.3408 469.5773 -215.6704 2 vs 3 4.636325 0.0313

lme.SRP.Treat.Day.Inter 4 13 435.9732 465.2036 -204.9866 3 vs 4 21.367670 0.0003

Fixed effects: SRP ~ Treatment + Day + Treatment:Day

Value Std.Error DF t-value p-value

(Intercept) 5.729932 0.9025430 30 6.348652 0.0000

TreatmentMQPvsRWP 3.488662 1.6478106 30 2.117150 0.0426

TreatmentMQvsMQP 4.185941 1.6478106 30 2.540305 0.0165

TreatmentARTvsPIT -1.663568 0.6016954 30 -2.764801 0.0096

TreatmentESTvsAGE 2.665608 0.6016954 30 4.430163 0.0001

Day42 -2.404834 0.8597279 30 -2.797204 0.0089

TreatmentMQPvsRWP:Day42 -2.529521 1.5696412 30 -1.611528 0.1175

TreatmentMQvsMQP:Day42 -0.449071 1.5696412 30 -0.286098 0.7768

TreatmentARTvsPIT:Day42 0.985709 0.5731519 30 1.719804 0.0958

TreatmentESTvsAGE:Day42 -2.686071 0.5731519 30 -4.686490 0.0001

#####################################################################################

---------------------NO3----------------------------

Model df AIC BIC LogLik Test L. Ratio p-value

lme.NO3 1 4 384.5276 393.5216 -188.2638

lme.NO3.Treat 2 8 380.7305 398.7185 -182.3653 1 vs 2 11.797110 0.0189

lme.NO3.Treat.Day 3 9 380.4163 400.6528 -181.2081 2 vs 3 2.314194 0.1282

lme.NO3.Treat.Day.Inter 4 13 359.7608 388.9913 -166.8804 3 vs 4 28.655461 <.0001

Fixed effects: NO3 ~ Treatment + Day + Treatment:Day

Value Std.Error DF t-value p-value

(Intercept) 3.487430 0.5389317 30 6.471005 0.0000

TreatmentMQPvsRWP 0.377474 0.9839502 30 0.383631 0.7040

TreatmentMQvsMQP 1.099987 0.9839502 30 1.117929 0.2725

TreatmentARTvsPIT 1.929122 0.3592878 30 5.369294 0.0000

TreatmentESTvsAGE -0.504538 0.3592878 30 -1.404272 0.1705

Day42 1.023625 0.4746918 30 2.156400 0.0392

TreatmentMQPvsRWP:Day42 0.901539 0.8666646 30 1.040240 0.3065

TreatmentMQvsMQP:Day42 0.769133 0.8666646 30 0.887464 0.3819

TreatmentARTvsPIT:Day42 -1.908637 0.3164612 30 -6.031187 0.0000

TreatmentESTvsAGE:Day42 0.668791 0.3164612 30 2.113343 0.0430

#############################################################################

------------------------------------NH4-----------------------------------

Model df AIC BIC LogLik Test L. Ratio p-value

lme.NH4 1 4 405.3686 414.3625 -198.6843

lme.NH4.Treat 2 8 407.7455 425.7334 -195.8727 1 vs 2 5.623097 0.2291

lme.NH4.Treat.Day 3 9 408.9018 429.1383 -195.4509 2 vs 3 0.843653 0.3584

lme.NH4.Treat.Day.Inter 4 13 407.5855 436.8160 -190.7928 3 vs 4 9.316269 0.0537

Fixed effects: NH4 ~ Treatment + Day + Treatment:Day

Value Std.Error DF t-value p-value

(Intercept) 5.131148 0.6944761 30 7.388516 0.0000

TreatmentMQPvsRWP 0.103498 1.2679342 30 0.081628 0.9355

TreatmentMQvsMQP 0.018948 1.2679342 30 0.014944 0.9882

TreatmentARTvsPIT -0.357785 0.4629841 30 -0.772781 0.4457

TreatmentESTvsAGE 1.187149 0.4629841 30 2.564124 0.0156

Day42 -0.784081 0.8022877 30 -0.977307 0.3362

TreatmentMQPvsRWP:Day42 -1.048788 1.4647702 30 -0.716009 0.4795

TreatmentMQvsMQP:Day42 1.304264 1.4647702 30 0.890422 0.3803

TreatmentARTvsPIT:Day42 -0.461078 0.5348584 30 -0.862056 0.3955

TreatmentESTvsAGE:Day42 -1.133566 0.5348584 30 -2.119376 0.0424

#############################################################################

#############################################################################

-------------------Cell Abundance----------------------

Model df AIC BIC LogLik Test L. Ratio p-value lme.Cells 1 4 1340.877 1354.766 -666.4387

lme.Cells.Group 2 8 1334.716 1362.494 -659.3579 1 vs 2 14.16160 0.0068

lme.Cells.Group.Time 3 14 1272.498 1321.109 -622.2488 2 vs 3 74.21823 <.0001

lme.Cells.Group.Time.Inter 4 38 1275.911 1407.857 -599.9553 3 vs 4 44.58702 0.0065

Value Std.Error DF t-value p-value

(Intercept) 6.227368 0.3035082 172 20.517959 0.0000

GroupMQPvsRWP 0.092462 0.5566426 30 0.166107 0.8692

GroupMQvsMQP 1.202634 0.5597590 30 2.148486 0.0399

GroupARTvsPIT -0.306278 0.2009646 30 -1.524040 0.1380

GroupESTvsAGE 0.572374 0.2007714 30 2.850873 0.0078

Timepoint.7 1.992560 0.5444884 172 3.659509 0.0003

Timepoint.14 -3.133829 0.5415874 172 -5.786377 0.0000

Timepoint.28 1.076064 0.5329905 172 2.018918 0.0450

Timepoint^55 -0.105738 0.5257266 172 -0.201128 0.8408

Timepoint^121 -2.962852 0.5206990 172 -5.690144 0.0000

Timepoint^314 -1.290647 0.5183632 172 -2.489851 0.0137

GroupMQPvsRWP:Timepoint.7 1.885645 1.0186228 172 1.851170 0.0659

GroupMQvsMQP:Timepoint.7 0.165600 1.0451736 172 0.158443 0.8743

GroupARTvsPIT:Timepoint.7 -0.050928 0.3476898 172 -0.146475 0.8837

GroupESTvsAGE:Timepoint.7 0.345518 0.3497841 172 0.987804 0.3246

GroupMQPvsRWP:Timepoint.14 1.185648 1.0133913 172 1.169981 0.2436

GroupMQvsMQP:Timepoint.14 -0.669117 1.0381226 172 -0.644545 0.5201

GroupARTvsPIT:Timepoint.14 0.493009 0.3447927 172 1.429870 0.1546

GroupESTvsAGE:Timepoint.14 -1.077092 0.3492833 172 -3.083719 0.0024

GroupMQPvsRWP:Timepoint.28 0.466412 0.9841211 172 0.473938 0.6361

GroupMQvsMQP:Timepoint.28 0.544861 0.9984521 172 0.545705 0.5860

GroupARTvsPIT:Timepoint.28 0.017411 0.3496162 172 0.049800 0.9603

GroupESTvsAGE:Timepoint.28 -0.438782 0.3478109 172 -1.261555 0.2088

GroupMQPvsRWP:Timepoint^55 -1.565231 0.9592569 172 -1.631712 0.1046

GroupMQvsMQP:Timepoint^55 -0.571130 0.9644359 172 -0.592191 0.5545

GroupARTvsPIT:Timepoint^55 0.501368 0.3534131 172 1.418645 0.1578

GroupESTvsAGE:Timepoint^55 -0.274036 0.3465803 172 -0.790687 0.4302

GroupMQPvsRWP:Timepoint^121 0.011804 0.9483649 172 0.012447 0.9901

GroupMQvsMQP:Timepoint^121 -0.379350 0.9494343 172 -0.399553 0.6900

GroupARTvsPIT:Timepoint^121 0.481500 0.3501064 172 1.375295 0.1708

GroupESTvsAGE:Timepoint^121 -0.947871 0.3457359 172 -2.741605 0.0068

GroupMQPvsRWP:Timepoint^314 1.559145 0.9458139 172 1.648469 0.1011

GroupMQvsMQP:Timepoint^314 -1.181952 0.9459114 172 -1.249538 0.2132

GroupARTvsPIT:Timepoint^314 0.024045 0.3462568 172 0.069443 0.9447

GroupESTvsAGE:Timepoint^314 0.149176 0.3453457 172 0.431962 0.6663

#############################################################################

#############################################################################

All DNA data used in this study can be found archived as zipped fastq files on figshare through the following link:

<https://figshare.com/articles/preprint/GH_MS_Sequence_Archive_zip/16735096>
